# High-Throughput Proteomics Identifies Proteins With Importance to Postantibiotic Recovery in Depolarized Persister Cells

**DOI:** 10.3389/fmicb.2019.00378

**Published:** 2019-03-06

**Authors:** Daniel-Timon Spanka, Anne Konzer, Daniel Edelmann, Bork A. Berghoff

**Affiliations:** ^1^Institute for Microbiology and Molecular Biology, Justus Liebig University Giessen, Giessen, Germany; ^2^Biomolecular Mass Spectrometry, Max-Planck-Institute for Heart and Lung Research, Bad Nauheim, Germany

**Keywords:** persister cells, antibiotic tolerance, depolarization, TisB toxin, recovery, SILAC, proteomics

## Abstract

Bacterial populations produce phenotypic variants called persisters to survive harmful conditions. Persisters are highly tolerant to antibiotics and repopulate environments after the stress has vanished. In order to resume growth, persisters have to recover from the persistent state, but the processes behind recovery remain mostly elusive. Deciphering these processes is an essential step toward understanding the persister phenomenon in its entirety. High-throughput proteomics by mass spectrometry is a valuable tool to assess persister physiology during any stage of the persister life cycle, and is expected to considerably contribute to our understanding of the recovery process. In the present study, an *Escherichia coli* strain, that overproduces the membrane-depolarizing toxin TisB, was established as a model for persistence by the use of high-throughput proteomics. Labeling of TisB persisters with stable isotope-containing amino acids (pulsed-SILAC) revealed an active translational response to ampicillin, including several RpoS-dependent proteins. Subsequent investigation of the persister proteome during postantibiotic recovery by label-free quantitative proteomics identified proteins with importance to the recovery process. Among them, AhpF, a component of alkyl hydroperoxide reductase, and the outer membrane porin OmpF were found to affect the persistence time of TisB persisters. Assessing the role of AhpF and OmpF in TisB-independent persisters demonstrated that the importance of a particular protein for the recovery process strongly depends on the physiological condition of a persister cell. Our study provides important insights into persister physiology and the processes behind recovery of depolarized cells.

## Introduction

The rise of antibiotic resistance among pathogens is a major threat to the human health care system (Lewis, 2013), and gains ever-expanding attention. Bacteria have, however, developed alternative strategies to survive an antibiotic challenge. For instance, Joseph W. Bigger realized very early after the introduction of penicillin that a small subpopulation of otherwise susceptible *Staphylococcus aureus* cultures survived a penicillin treatment for several days. He termed these surviving cells “persisters” (Bigger, 1944). Persisters are transiently drug-tolerant phenotypic variants within isogenic populations and, in contrast to resistant bacteria, do not proliferate in the presence of antibiotics. Furthermore, the minimum inhibitory concentration (MIC) for a persistent strain is not altered in comparison to a strain that is susceptible to a particular antibiotic (Brauner et al., 2016). Even though persisters have been extensively studied during the last years, there is still a lack of knowledge regarding the physiological state of persisters. The general perception of persisters is that they are non-growing or slowly growing cells (Balaban et al., 2004), and that reduced activity of antibiotic targets renders them tolerant to antibiotics (Lewis, 2010). Clearly, different types of persisters exist (Balaban et al., 2004), and it is feasible to assume that physiological states are different as well. For example, extremely dormant persisters might be similar to viable but non-culturable (VBNC) cells (Ayrapetyan et al., 2015; Kim et al., 2018). By contrast, persisters induced by carbon source shifts retain metabolic activity, exhibit slow growth, and are marked by a distinct proteome pattern (Radzikowski et al., 2016). Hypothetically, the physiology of persisters is dependent on the particular mechanism that has triggered entry into the persistent state.

Endogenous factors, that reduce cellular activity and potentially favor persistence, are toxins from toxin-antitoxin (TA) systems. TA systems were discovered on plasmids, where they are implicated in plasmid maintenance during cell proliferation, but were later also identified on bacterial chromosomes in surprisingly high numbers (Hayes, 2003; Gerdes et al., 2005). They are classified according to the specific mechanism by which the antitoxin inhibits expression or activity of its toxin counterpart (Page and Peti, 2016). In type I TA systems, the antitoxin is an antisense RNA that specifically blocks translation of the toxin mRNA (Fozo et al., 2008a; Brantl and Jahn, 2015). In type II TA systems, the antitoxin inhibits activity of the toxin via protein-protein interaction (Gerdes and Maisonneuve, 2012). Several lines of evidence indicate that chromosomal TA systems play a role in persister formation. In fact, the first “persister gene” to be discovered was *hipA* from the type II TA system *hipAB* in *E. coli* (Moyed and Bertrand, 1983; Black et al., 1991, 1994). Toxin HipA inactivates glutamyl-tRNA-synthetase (GltX) by phosphorylation, which leads to disturbed aminoacylation (Germain et al., 2013; Kaspy et al., 2013). As a consequence, the stringent response alarmone (p)ppGpp is produced and transition into the persistent state is favored (Korch et al., 2003). The possible role of chromosomal TA systems in bacterial persistence was further underscored in the mid 2000's, when it was observed that several toxin genes from type II TA systems were upregulated in persister cells, which for example applies to *mazF* and *relE* (Keren et al., 2004; Shah et al., 2006). MazF and RelE are mRNA endonucleases that impede translation and cause growth stasis. Intriguingly, both activation of MazF and overexpression of RelE mediated persister formation in *E. coli* (Keren et al., 2004; Tripathi et al., 2014). Further evidence for toxins as “persistence factors” comes from work with the mRNA endonucleases YafQ and MqsR. Single gene deletions of *yafQ* and *mqsR* caused a reduction in persistence (Harrison et al., 2009; Kim and Wood, 2010). Finally, toxins from type I TA systems have been directly linked to persister formation. These toxins are often small hydrophobic proteins that preferentially localize to the inner membrane to cause break-down of the proton motive force or ATP leakage (Fozo et al., 2008b; Unoson and Wagner, 2008; Gurnev et al., 2012; Weel-Sneve et al., 2013; Wilmaerts et al., 2018). Membrane depolarization and depletion of intracellular ATP potentially trigger entry into a persistent state, as shown for toxins TisB and HokB (Dörr et al., 2010; Verstraeten et al., 2015; Berghoff et al., 2017; Wilmaerts et al., 2018). In *E. coli* and *S. aureus*, artificial ATP depletion by the addition of arsenate is sufficient to induce persister formation (Conlon et al., 2016; Shan et al., 2017). However, membrane depolarization alone, rather than ATP depletion, might be a determinant of persistence in *S. aureus* as well (Wang et al., 2018).

Another central question in the persister field concerns the mechanisms that affect persister awakening. The “microbial scout” model suggests that dormant cells awake stochastically to sample their environment for suitable conditions (Buerger et al., 2012). In case of *Bacillus* spores, the model seems reasonable, and some spores were indeed shown to awaken spontaneously without sensing of outside signals (Sturm and Dworkin, 2015). Work with the model bacterium *E. coli* showed that the outgrowth medium has an influence on wake-up kinetics of persister cells (Jõers et al., 2010), indicating that persister cells may sense their environment. It is, however, not known which cellular proteins are at the forefront of awakening. Do persisters express specific proteins to neutralize the effect of a particular toxin? The answer is possibly yes; it was demonstrated that acetylation of tRNAs by toxin TacT is reversed by a peptidyl-tRNA hydrolase (Cheverton et al., 2016). However, this specific enzyme cannot explain awakening of persisters that have formed through other mechanisms. Moreover, even though neutralization of toxins might initiate awakening, it can be expected that further proteins serve specific functions during the subsequent recovery process. For instance, DNA repair during recovery seems to be key to persistence of non-growing *E. coli* cells after ofloxacin treatment (Völzing and Brynildsen, 2015).

In the present study, we have chosen TisB as a model system for “persistence by depolarization” during exponential growth phase (Dörr et al., 2010; Berghoff et al., 2017). TisB is the toxin moiety of the TisB/IstR-1 TA system and is induced upon DNA damage due to activation of the SOS response via cleavage of the LexA repressor (Vogel et al., 2004). TisB targets the inner membrane and causes depolarization (Unoson and Wagner, 2008; Gurnev et al., 2012). Translation of the primary *tisB* mRNA is repressed by an inhibitory secondary structure in its 5′ untranslated region (UTR). After cleavage of the 5′ UTR structure, translation is blocked by the antitoxin IstR-1 (Darfeuille et al., 2007; Berghoff and Wagner, 2017). We recently deleted both regulatory RNA elements in *E. coli* K-12 wild type MG1655. The resulting double deletion strain Δ1-41 Δ*istR* exhibits unchecked expression of TisB, is highly persistent when treated with different antibiotics, and represents, therefore, a suitable system to study TisB-dependent persisters (Berghoff et al., 2017). Here, state-of-the-art mass spectrometry (MS) methods were applied to assess the persister proteome, both during antibiotic challenge and during postantibiotic recovery. A pulsed-SILAC (stable isotope labeling by amino acids in cell culture) approach was applied during ampicillin treatment, highlighting 43 proteins with significantly increased protein synthesis. Since many of these proteins are stress-related and serve protective functions, we conclude that TisB-dependent persisters mount an active response to ampicillin. Furthermore, protein samples from the recovery phase were analyzed by label-free quantitative MS to identify proteins with differential abundance. Among the 24 proteins with increased abundance during recovery, we identified a component of the alkyl hydroperoxide reductase (AhpF) and an outer membrane porin (OmpF). Deletions of *ahpF* and *ompF* in the Δ1-41 Δ*istR* background caused an extended period of persistence after antibiotic treatment, indicating that both proteins play important roles during recovery from the persistent state. However, subsequent experiments showed that these functions are specific to TisB-dependent persister cells. We conclude that functions needed for the recovery process have to match the specific physiological state of a persister cell.

## Materials and Methods

### Growth Conditions

For physiological experiments, *E. coli* strains were grown in lysogeny broth (LB) or M9 minimal medium at 37°C with continuous shaking at 180 rpm (aerobic growth). *E. coli* strains containing temperature-sensitive plasmids were grown at 30°C. If applicable, antibiotics were added at the following concentrations: 50 μg ml^−1^ kanamycin, 15 μg ml^−1^ chloramphenicol, 6 μg ml^−1^ tetracycline, 50 μg ml^−1^ ampicillin. Over-night cultures were diluted 100-fold into fresh medium and incubated until an optical density at 600 nm (OD_600_) of 0.3–0.6 (exponential phase) was reached. For stationary phase experiments, liquid cultures were inoculated with single colonies and grown for 20 h. For growth curves, the initial OD_600_ was adjusted to 0.02. Growth curves were monitored using a Cell density meter model 40 (Fisher Scientific). Doubling times were calculated from exponential growth phase and *P*-values were assessed using Student's *t*-test.

### Construction of Bacterial Strains

*E. coli* strains used in this study are derivatives of K-12 wild type MG1655 and are listed in Supplementary Table 1. Chromosomal deletions of candidate genes were constructed by homologous recombination using the λ red genes (Yu et al., 2000). A chloramphenicol acetyltransferase (*cat*) or kanamycin resistance (*kan*) gene was PCR-amplified together with specific overhangs (40 bp) on each side to enable recombination within the desired gene locus. The corresponding oligodeoxyribonucleotides are listed in Supplementary Table 2. The linear amplification products were transformed into *E. coli* strains containing temperature-sensitive plasmid pSIM5 for heat-inducible expression of λ red genes (Datta et al., 2006). After recombination, clones were selected on LB agar plates containing chloramphenicol (12.5 μg ml^−1^) or kanamycin (25 μg ml^−1^), respectively. Insertion of the *cat* or *kan* gene was verified by PCR with oligodeoxyribonucleotides listed in Supplementary Table 2. Deletion constructs containing selectable markers were transferred to the wild-type background by P1 transduction.

For simultaneous deletion of *ahpF* and *ompF* in strain B133 (Δ1-41 Δ*istR::frt-kan-frt*), the *kan* gene of strain B133 was removed by FLP-mediated site-directed mutagenesis using plasmid 709-FLPe (Gene Bridges) according to the manufacturer's protocol. Clones were tested for kanamycin sensitivity, and *ahpF* and *ompF* were subsequently deleted by λ red-mediated recombination using *cat* and *kan* genes, respectively, for selection. The corresponding oligodeoxyribonucleotides are listed in Supplementary Table 2.

### Pulse-Labeling With Stable Isotopes

*E. coli* strain B133 (Δ1-41 Δ*istR::frt-kan-frt*) was pulse-labeled with the stable isotope-containing amino acid L-lysine-^13^C_6_,^15^N_2_ (Lys8). The protocol is a combination of native and pulsed-SILAC approaches that have been successfully applied to prototrophic bacteria (Michalik et al., 2012; Berghoff et al., 2013; Fröhlich et al., 2013). *E. coli* was cultivated in M9 minimal medium (final concentrations: 47.7 mM Na_2_HPO_4_, 22 mM KH_2_PO_4_, 8.6 mM NaCl, 18.7 mM NH_4_Cl, 2 mM MgSO_4_, 0.1 mM CaCl_2_, 1 μg ml^−1^ thiamine) containing 0.4% glucose as carbon source (M9+glu). Regular L-lysine (Lys0) was added to over-night cultures at a final concentration of 30 μg ml^−1^. For pulse-labeling experiments, Erlenmeyer flasks (50 ml) were filled with 10 ml M9+glu, supplemented with 30 μg ml^−1^ Lys0, and inoculated with cells from over-night cultures. When an OD_600_ of 0.3–0.4 was reached, cells were pelleted by centrifugation (10,000 g, 3 min), washed in 1 ml M9+glu to remove Lys0, resuspended in 10 ml fresh M9+glu, and transferred to Erlenmeyer flasks (50 ml). Lys8 (30 μg ml^−1^) and ampicillin (200 μg ml^−1^) were immediately added to start pulse-labeling and antibiotic challenge in parallel. A culture incubated in the presence of Lys8 but without ampicillin served as treatment control. Cultures were incubated for 4 h at 37°C and 180 rpm. Cells were harvested by cold centrifugation (10,000 g, 3 min, 4°C), washed with 1 ml ice-cold M9+glu, and pelleted by centrifugation (10,000 g, 3 min, 4°C). All cell pellets were stored at −20°C until preparation of protein samples for mass spectrometry analysis.

### Recovery Experiments

Recovery experiments were performed in biological triplicates with *E. coli* strain B133 (Δ1-41 Δ*istR::frt-kan-frt*). Erlenmeyer flasks (100 ml) were filled with 20 ml LB medium and inoculated with cells from over-night cultures. When exponential phase (OD_600_ 0.3–0.6) was reached, cultures were treated with 200 μg ml^−1^ ampicillin to lyse non-persistent cells. Persister cells were harvested by centrifugation (10,000 g, 3 min) after 2 h of treatment. Cells were either washed with 1 ml ice-cold NaCl solution (0.9%) and pelleted by centrifugation (10,000 g, 3 min, 4°C) or prepared for recovery in fresh LB medium without antibiotics. For this purpose, cells were washed in 1 ml NaCl solution (0.9%), followed by centrifugation (10,000 g, 3 min), resuspension in 20 ml LB medium, and incubation in Erlenmeyer flasks (100 ml) at 37°C and 180 rpm. At different time points during and following recovery, cells were harvested by cold centrifugation and washed with ice-cold NaCl solution (0.9%) as described before. All cell pellets were stored at −20°C until preparation of protein samples for SDS-PAGE or mass spectrometry analysis.

### SDS-PAGE of Protein Samples

Cell pellets from recovery experiments were resuspended in 500 μl phosphate buffer (50 mM, pH 7.2) and disrupted by sonication on ice (3 × 30 s; cycle: 70%; power: 70%). Cell debris and intact cells were removed by centrifugation (13,000 rpm, 10 min, 4°C). Protein concentration of supernatants was determined using a NanoDrop ND-1000 spectrophotometer (PeqLab). Two hundred micrograms of protein were precipitated with acetone at −20°C for 3 h. After centrifugation (13,000 rpm, 10 min, 4°C), protein pellets were washed two times with ice-cold acetone. Pellets were resolved in 1 × SDS sample buffer at 95°C for 10 min, and 50 μg of protein were loaded on 12% polyacrylamide gels. Gels were stained with colloidal Coomassie (Roth) for visualization of protein bands.

### Protein Sample Preparation for Mass Spectrometry

*E. coli* cell pellets were lysed in SDS-buffer (4% SDS in 0.1 M Tris/HCl, pH 7.6) by heating at 70°C for 10 min and sonication. Next, solubilized proteins were separated from cell debris by centrifugation at 16,000 g for 10 min and protein concentration in the supernatant was determined using the DC protein assay (BioRad). From each sample, an equal protein amount was precipitated by 4 volumes of 100% acetone at −20°C for 1 h, pelleted at 14,000 g for 10 min and washed with 90% acetone. Dried protein pellets were dissolved in urea buffer (6 M urea, 2 M thiourea, 10 mM HEPES, pH 8.0) and enzymatic protein digest was performed by in-solution digestion as previously described (Andersen et al., 2005). In brief, protein disulfide bonds were reduced with 10 mM dithiothreitol and alkylated with 55 mM iodoacetamide. Next, proteins were cleaved enzymatically in two steps at room temperature: pre-digestion was performed by Lys-C (100:1 protein-to-enzyme ratio) (Wako Chemicals GmbH) for 3 h followed by an overnight treatment with Trypsin (100:1 protein-to-enzyme ratio) (Serva). Finally, resulting peptides were desalted by stop and go extraction (STAGE) tips (Rappsilber et al., 2003) before LC-MS/MS analysis.

### Mass Spectrometry

LC-MS/MS analysis was performed using an UHPLC system (EASY-nLC 1000, Thermo Fisher Scientific) and a QExactive HF Orbitrap mass spectrometer (Thermo Fisher Scientific) as already described (Worzfeld et al., 2018). The same parameters were used except for the gradient of the reverse-phase chromatography: peptides were separated using a linearly increasing concentration of solvent B (80% acetonitrile, 0.1% formic acid) over solvent A (0.1% formic acid) from 5 to 30% for 215 min and from 30 to 60% for 5 min, followed by washing with 95% of solvent B for 5 min and re-equilibration with 5% of solvent B.

### Evaluation of Proteome Data

MS raw data were processed by MaxQuant (1.5.6.5) (Cox and Mann, 2008) and the implemented Andromeda search engine using a Uniprot database of *E. coli* (strain K12) containing 4,306 entries (release 2017-01). The following parameters were used for data processing: maximum of two miss cleavages and mass tolerance of 4.5 ppm for main search. Trypsin was set as digesting enzyme. As fixed modification we used carbamidomethylation of cysteins and variable modifications were defined as oxidation of methionine and acetylation of the protein N-terminus. Beside these default parameters of MaxQuant, “label-free quantification” (LFQ) was enabled for protein quantification. For downstream data analysis, only proteins with at least two peptides and at least one unique peptide were considered as identified and processed with Perseus (1.5.6.0) to calculate *P*-values based on Benjamini–Hochberg multiple testing correction with a FDR threshold of 0.05. Processed data were evaluated using R statistical language (http://www.r-project.org/) and DAVID bioinformatics database (Huang et al., 2009) (https://david.abcc.ncifcrf.gov/home.jsp). Principal component analysis (PCA) was applied to LFQ intensities (log_2_) using R function *prcomp*. Package “factoextra” was used for visualization of PCA plots. Significantly regulated proteins were clustered according to functional annotations using the DAVID bioinformatics database. Gene ontology (GO) terms BP (biological process), CC (cellular component), MF (molecular function), KEGG pathway information (https://www.genome.jp/kegg/), and InterPro protein domains (https://www.ebi.ac.uk/interpro/) were selected to identify enriched clusters. The proteome data can be found in Supplementary Table 3.

### Persister Assays

Persister levels were calculated by plating serial dilutions of cultures before and after antibiotic treatment. Exponential phase cultures (OD_600_ 0.3–0.6) were treated with 200 μg ml^−1^ ampicillin or 10 μg ml^−1^ ciprofloxacin, and subsequently incubated for 3 h at 37°C and 180 rpm. Stationary phase cultures (20 h after inoculation) were treated with 10 μg ml^−1^ ciprofloxacin for 5 h. Serial dilutions were prepared with NaCl solution (0.9%) and plated on LB agar without antibiotics. Agar plates were incubated at 37°C and colonies either counted after ~20 h (before treatment) or ~40 h (after treatment). Colony counts were used to calculate colony forming units (CFU) per milliliter. The persister level was the ratio between CFU ml^−1^ from treated samples and CFU ml^−1^ from untreated samples. *P*-values were calculated using Student's *t*-test.

### ScanLag Analysis

Colony growth on LB agar plates was monitored using the ScanLag method (Levin-Reisman et al., 2010, 2014). Agar plates were covered with black felt and placed on Epson Perfection V39 scanners to record time series of images using the *ScanningManager* application. The equipment was placed in a 37°C incubation room. Images (stored as ^*^.*tif* files) were recorded every 20 min for a total time period of 40 h. Images were processed in MatLab (MathWorks) using functions *PreparePictures, setMaskApp*, and *TimeLapse*. Function *ScanLagApp* was called to assess the quality of single colonies. Afterwards, appearance and growth times were extracted from the data. The appearance time of a colony equals the time point at which the colony has a minimum size of 10 pixels. The growth time of a colony is defined as the time needed to increase in size from 80 to 160 pixels. Growth inhibition by neighboring colonies can produce late appearance of individual colonies on densely populated plates (>100 colonies), which would falsely skew distribution plots toward late appearance times. In most cases, these colonies exhibit extraordinarily long growth times as well. Therefore, raw data were corrected based on the interquartile range of growth times: the upper limit *L* was defined as *L* = *Q*_3_ + 1.5 ^*^ (*Q*_3_ – *Q*_1_), with *Q*_1_ and *Q*_3_ being the lower and upper quartile, respectively. All colonies with growth times above *L* were removed from the analysis. For statistical evaluation of growth parameters R statistical language (http://www.r-project.org/) was used. Shapiro–Wilk test (function *shapiro.test* from package “stats”) was called to analyze the distribution of appearance and growth times, demonstrating that the data were not normally distributed. Mann–Whitney-Wilcoxon test (function *wilcox.test* from package “stats”) was subsequently used to calculate *P*-values.

## Results

### Assessing the Persister Proteome by a Pulsed-SILAC Approach

**Figure 1:**
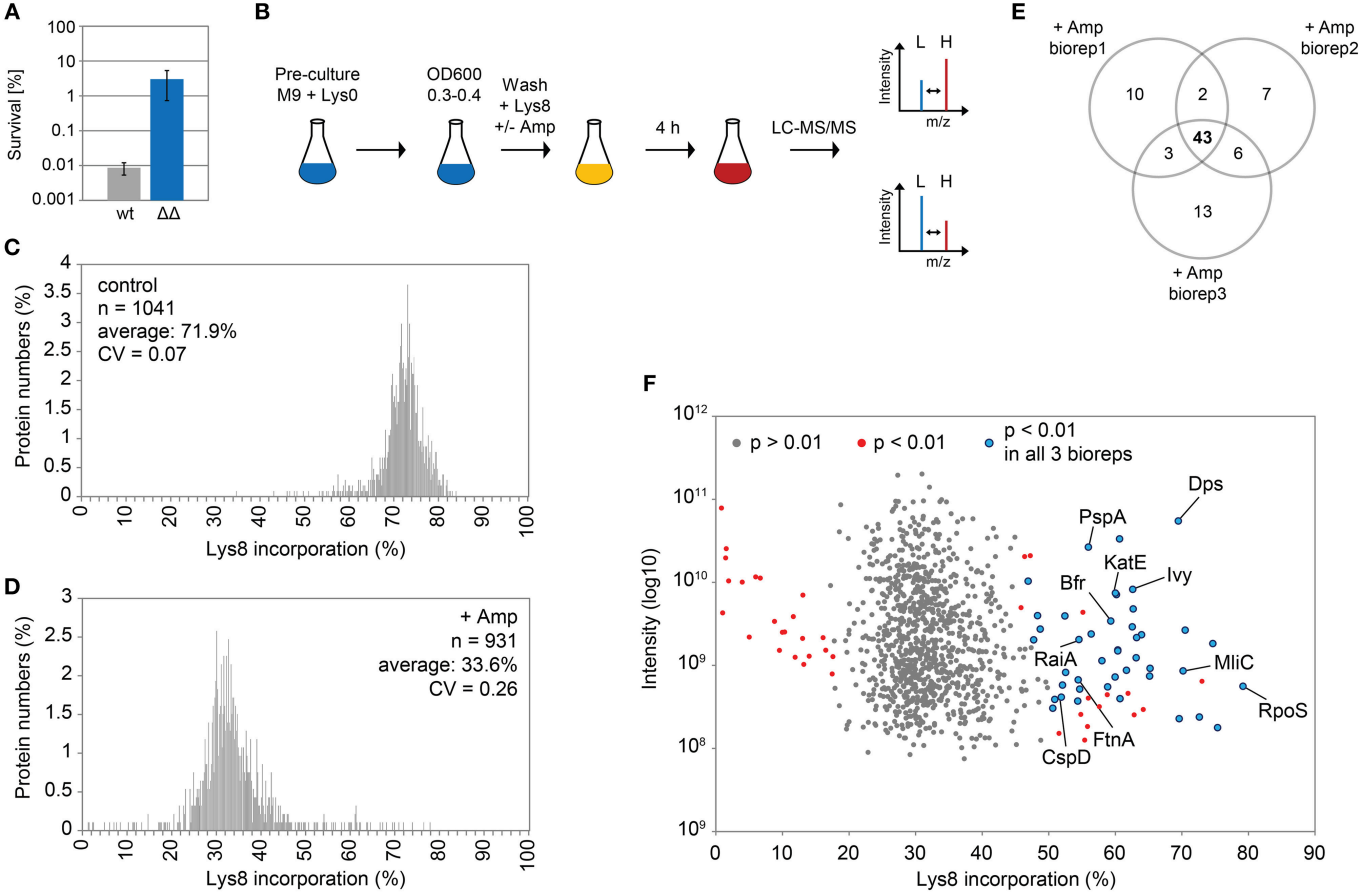
Pulsed-SILAC during ampicillin treatment reveals newly synthesized proteins. **(A)** Persister levels after 4 h of ampicillin treatment (200 μg ml^−1^) during exponential growth phase in M9+glu. CFU counts before and after ampicillin treatment were used to calculate survival. Data represent the mean of at least three independent biological replicates for wild type MG1655 (wt) and Δ1-41 Δ*istR* (ΔΔ). Error bars depict standard deviations. **(B)** Illustration of the pulsed-SILAC approach. Pre-cultures were grown in M9+glu in the presence of regular L-lysine (Lys0; 30 μg ml^−1^). When exponential growth phase was reached, Lys0 was removed by washing, and cultures were incubated in the presence of L-lysine-^13^C_6_, ^15^N_2_ (Lys8; 30 μg ml^−1^) and ampicillin (200 μg ml^−1^) for 4 h. A control culture was treated with Lys8, but allowed to grow in the absence of ampicillin. Protein samples were analyzed by LC-MS/MS to determine heavy (H) to light (L) protein ratios. H/L ratios were subsequently used to calculate Lys8 incorporation in percent. **(C)** Distribution plot of Lys8 incorporation (%) for the control culture. The number of quantified proteins (*n*), the average Lys8 incorporation and coefficient of variation (CV) are indicated. **(D)** Distribution plot of Lys8 incorporation (%) for ampicillin-treated cultures. Data represent the average of three biological replicates (Supplementary Figure 1). The number of quantified proteins (*n*), the overall average of Lys8 incorporation and coefficient of variation (CV) are indicated. **(E)** Venn diagram depicting proteins with significantly increased Lys8 incorporation (Significance B analysis, *P* < 0.01). Data were derived from ampicillin treatments in three biological replicates (biorep1-3). **(F)** Illustration of Significance B analysis for biorep1. Red dots represent proteins with a significantly altered (*P* < 0.01) Lys8 incorporation (%). Blue dots: *P* < 0.01 in all three biological replicates. Gray dots: *P* > 0.01.

Ampicillin and other β-lactam antibiotics cause rapid lysis of actively growing cells due to inhibition of cell wall biosynthesis. A treatment with ampicillin will, therefore, efficiently eliminate the majority of cells in exponential cultures of ampicillin-sensitive bacteria, and is considered as a simple method to enrich persister fractions (Keren et al., 2004). Here, we treated the highly persistent *E. coli* strain Δ1-41 Δ*istR* with a high dose of ampicillin (200 μg ml^−1^) during exponential growth phase (OD_600_ ~0.3) in M9 minimal medium, a situation in which up to 3% persister cells are formed (Figure 1A). Since wild-type cultures produce >300-fold less persisters (Figure 1A), the vast majority of persister cells in strain Δ1-41 Δ*istR* can be expected to depend on TisB-induced depolarization for their generation (Berghoff et al., 2017). In parallel to the ampicillin challenge, pulse-labeling with the stable isotope-containing amino acid L-lysine-^13^C_6_,^15^N_2_ (Lys8) was performed to assess its incorporation into newly synthesized proteins during persister formation. Since our *E. coli* strains are prototrophic, pre-cultures were incubated in the presence of regular L-lysine (Lys0) to prime uptake and utilization of the externally added amino acid (Fröhlich et al., 2013). Lys0 was washed out the medium just before pulse-labeling with Lys8 was started (Figure 1B). After 4 h of ampicillin challenge and pulse-labeling, persister cells were harvested and analyzed by LC-MS/MS to determine heavy to light (H/L) protein ratios according to existing SILAC protocol (Schwanhäusser et al., 2009). H/L ratios were subsequently used to calculate Lys8 incorporation for the corresponding proteins. As a control, one culture was pulse-labeled with Lys8, but allowed to grow in the absence of ampicillin (Figure 1B). The control experiment demonstrated efficiency of the pulse-labeling protocol, as judged from the average Lys8 incorporation of 71.9% after 4 h of labeling (Figure 1C). By contrast, Lys8 incorporation in the ampicillin-treated persister cells was clearly reduced in all three biological replicates, with average values ranging from 32.8 to 34.4% (Supplementary Figure 1). Since reproducibility of Lys8 incorporation between replicates was high (Pearson's Rho ≥ 0.95; Supplementary Figure 2), data were combined in a single distribution plot displaying an overall average Lys8 incorporation of 33.6% for 931 proteins quantified in all biological replicates (Figure 1D). Notably, the variability of Lys8 incorporation, as measured by the coefficient of variation (CV), was considerably higher in persister cells (CV = 0.26) than in the control (CV = 0.07), indicating an increased heterogeneity in protein synthesis. The reduction of Lys8 incorporation of >2-fold compared to the control shows that ampicillin-treated persister cells are impaired in protein synthesis.

### TisB-Dependent Persister Cells Mount an Active Response to Ampicillin

We were especially interested in proteins that displayed a Lys8 incorporation significantly higher than the average, which was indicative of enhanced synthesis and functional importance upon ampicillin stress. For this purpose, Significance B analysis was performed for each biological replicate (Cox and Mann, 2008). A common group of 43 proteins with *P* < 0.01 emerged from all ampicillin experiments (Figures 1E,F; for details see Table 1). Only six of these proteins showed a significantly higher Lys8 incorporation in the control experiment, which applied to Cfa, Dps, LpxC, ClpA, RaiA, and YibT. *P*-values were however lower by several orders of magnitude in ampicillin experiments, with YibT being the only exception (Table 1). Functional annotation clustering using gene ontology (GO) terms, KEGG pathway information, and InterPro protein domains (Huang et al., 2009) failed to highlight any major functional category, except for a small cluster formed by FtnA, Bfr, and Dps. Ferritin (FtnA) and bacterioferritin (Bfr) are iron storage proteins, that bind major proportions of iron under both normal and high-iron conditions (Sevcenco et al., 2011). Dps is a multi-function protein involved in iron sequestration, detoxification of reactive oxygen species (ROS), and mechanical protection of DNA during stationary phase (Zeth, 2012). Interestingly, the ROS detoxifying enzyme KatE (catalase II) showed increased Lys8 incorporation as well. Transcription of both *dps* and *katE* is triggered by RpoS, the master regulator of the general stress response in *E. coli*. Intriguingly, RpoS had the highest Lys8 incorporation (77.7%) of all identified proteins (Table 1), indicating that the general stress response is switched on in TisB-dependent persister cells. Further proteins that are related to stationary phase were RaiA and CspD. RaiA (or protein Y) is a modulator of ribosome activity and associates with 70S ribosomes to inhibit translation initiation during cold shock and stationary phase (Vila-Sanjurjo et al., 2004). CspD is an inhibitor of DNA replication that is implicated in persister formation (Kim and Wood, 2010). CspD levels are high in stationary phase, but decline when growth resumes due to degradation by Lon protease (Langklotz and Narberhaus, 2011). Furthermore, the bifunctional protein PspA was found to exhibit an increased Lys8 incorporation. PspA inhibits its own transcriptional activator PspF under normal conditions. As soon as envelope stress occurs, PspA releases PspF and associates with PspBC to preserve functions of the inner membrane (Manganelli and Gennaro, 2017). Two other identified proteins, Ivy and MliC, are inhibitors of vertebrate C-type lysozyme and protect peptidoglycan against hydrolyzing attack. The above mentioned proteins are highlighted in a scatter plot that illustrates the Significance B analysis for one of the biological replicates (Figure 1F). The remaining proteins (Table 1) represent, e.g., periplasmic chaperones (OsmY, Spy), further RpoS-dependent proteins (cyclopropane-fatty-acyl-phospholipid synthase Cfa, trehalose-6-phosphate synthase OtsA, pyruvate dehydrogenase PoxB), proteins with functions in sulfate and sulfonate utilization [sulfate transporter subunit Sbp, sulfonate ABC transporter periplasmic binding protein SsuA, NAD(P)H-dependent FMN reductase SsuE], and proteins with a role in production of the capsular polysaccharide colanic acid (GDP-mannose 4,6-dehydratase Gmd, UDP-glucose 6-dehydrogenase Ugd). As a conclusion, our pulsed-SILAC approach identified 43 proteins with enhanced synthesis upon ampicillin stress, representing 4.6% of all quantified proteins. Many of these proteins serve stress-related and protective functions, and likely represent an active response of persister cells to ampicillin.

**Table 1:** Proteins with high Lys8 incorporation upon ampicillin treatment.

**Protein name**	**Lys8 incorporation (%)**					**Significance B (*****P*** **value)**				**Description**
	**Average**	**Biorep1**	**Biorep2**	**Biorep3**	**Control**	**Biorep1**	**Biorep2**	**Biorep3**	**Control**	
RpoS	77.69	79.17	76.59	77.32		1.95E-60	4.69E-45	4.99E-55		RNA polymerase sigma factor RpoS
YmgG	76.30	75.36	77.23	76.30		3.51E-37	7.10E-49	2.67E-48		UPF0757 protein YmgG
YgdI	73.65	70.50	75.48	74.97		1.22E-38	3.35E-39	6.13E-41		Uncharacterized lipoprotein YgdI
Cfa	71.69	74.66	67.07	73.35	80.44	9.39E-63	5.72E-15	1.28E-53	0.0067	Cyclopropane-fatty-acyl-phospholipid synthase
MliC	70.68	70.19	70.06	71.79		1.86E-20	8.52E-21	5.40E-28		Membrane-bound lysozyme inhibitor of C-type lysozyme
Ugd	69.71	69.60	70.81	68.71		3.51E-19	1.27E-22	1.02E-19		UDP-glucose 6-dehydrogenase
YcfJ	69.37	72.63	69.00	66.47		6.13E-27	1.76E-18	2.06E-15		Uncharacterized protein YcfJ
Dps	67.93	69.47	67.76	66.57	80.83	2.04E-34	9.35E-26	1.49E-24	0.0038	DNA protection during starvation protein
BioB	67.18	65.15	66.53	69.85		5.80E-12	4.00E-14	2.02E-22		Biotin synthase
YiaD	66.55	65.23	66.76	67.67		4.68E-12	1.77E-14	1.41E-17		Probable lipoprotein YiaD
LpxC	64.36	63.94	64.55	64.58	81.65	7.07E-19	4.43E-18	1.46E-12	8.81E-04	UDP-3-O-[3-hydroxymyristoyl] N-acetylglucosamine deacetylase
Ivy	63.65	62.62	64.65	63.70	77.86	2.13E-16	2.84E-18	7.11E-18	0.1016	Inhibitor of vertebrate lysozyme
Gmd	63.45	63.15	63.79	63.43		9.64E-10	1.46E-10	2.34E-17		GDP-mannose 4,6-dehydratase
TauA	62.15	63.21	61.20	62.02		1.81E-17	1.37E-12	7.00E-15		Taurine-binding periplasmic protein
SsuA	61.92	60.37	62.96	62.44		2.23E-07	1.09E-09	1.41E-15		Putative aliphatic sulfonates-binding protein
SsuE	61.36	60.71	60.56	62.80		1.25E-07	1.42E-07	2.15E-10		FMN reductase (NADPH)
LoiP	61.19	60.37	61.78	61.42	70.02	2.22E-07	1.41E-08	6.35E-14	0.5673	Metalloprotease LoiP
PlaP	61.14	57.99	61.61	63.82		7.61E-06	1.98E-08	1.37E-11		Low-affinity putrescine importer PlaP
YghA	61.08	62.56	60.08	60.60	78.35	2.70E-16	3.59E-11	9.81E-13	0.0687	Uncharacterized oxidoreductase YghA
OsmY	60.83	60.64	61.30	60.55	76.34	2.57E-13	9.93E-13	1.13E-12	0.2640	Osmotically-inducible protein Y
Spy	60.83	61.67	59.96	60.86	79.07	2.15E-08	4.01E-07	1.77E-08	0.0363	Spheroplast protein Y
ClpA	60.00	62.68	59.81	57.52	80.69	1.63E-16	7.60E-11	4.15E-09	0.0047	ATP-dependent Clp protease ATP-binding subunit ClpA
Bfr	59.89	59.32	60.61	59.74	57.94	1.33E-11	8.17E-12	1.34E-11	0.0179	Bacterioferritin
Sbp	59.68	60.17	59.34	59.52	74.90	1.13E-12	2.57E-10	2.50E-11	0.5276	Sulfate-binding protein
YbgS	59.52	59.97	59.42	59.15	79.39	4.27E-07	9.57E-07	4.22E-07	0.0259	Uncharacterized protein YbgS
KatE	58.49	60.02	57.97	57.49	74.35	1.75E-12	6.55E-09	4.43E-09	0.5987	Catalase HPII
ZntA	57.92	58.84	56.42	58.49		2.38E-06	5.39E-05	1.24E-06		Lead, cadmium, zinc and mercury-transporting ATPase
LipA	56.42	54.37	57.10	57.79		3.93E-04	2.42E-05	3.62E-06		Lipoyl synthase
PspA	56.08	55.96	56.38	55.90	76.82	3.59E-08	1.63E-07	1.21E-07	0.2154	Phage shock protein A
YebE	55.69	56.39	56.07	54.61		1.53E-08	2.86E-07	1.22E-06		Inner membrane protein YebE
YciE	54.48	54.65	55.14	53.65	76.85	3.03E-04	2.11E-04	4.79E-04	0.2119	DUF892 domain-containing protein YciE
GatZ	54.19	48.73	56.51	57.31		7.58E-04	4.86E-05	7.11E-06		D-tagatose-1,6-bisphosphate aldolase subunit GatZ
RaiA	53.93	54.53	52.91	54.35	80.98	4.84E-07	3.43E-05	1.87E-06	0.0029	Ribosome-associated inhibitor A
HemA	53.90	50.61	54.58	56.51		0.0065	3.64E-04	2.08E-05		Glutamyl-tRNA reductase
CspD	53.74	51.89	55.83	53.49	78.89	0.0028	1.04E-04	5.55E-04	0.0437	Cold shock-like protein CspD
YfdC	52.46	52.53	53.33	51.50		0.0018	0.0011	0.0029		Inner membrane protein YfdC
Ahr	52.25	52.07	52.60	52.09	61.94	0.0025	0.0019	0.0018	0.0576	Aldehyde reductase Ahr
ArgF	51.86	47.73	53.12	54.72	57.32	0.0018	2.61E-05	1.02E-06	0.0151	Ornithine carbamoyltransferase chain F
FtnA	51.69	54.44	49.24	51.41		3.69E-04	0.0017	0.0031		Bacterial non-heme ferritin
YibT	50.54	50.91	50.59	50.11	80.27	0.0054	0.0078	0.0076	0.0086	Uncharacterized protein YibT
PoxB	49.67	52.44	48.73	47.85	76.55	1.16E-05	0.0025	0.0037	0.2366	Pyruvate dehydrogenase [ubiquinone]; Alpha-peptide
OtsA	48.51	48.32	49.00	48.20	77.95	0.0011	0.0020	0.0028	0.1002	Alpha, alpha-trehalose-phosphate synthase [UDP-forming]
IlvB	48.01	46.91	47.53	49.58	60.54	0.0035	0.0064	8.03E-04	0.0381	Acetolactate synthase isozyme 1 large subunit

### Proteome Analysis of Recovering Persister Cells

**Figure 2:**
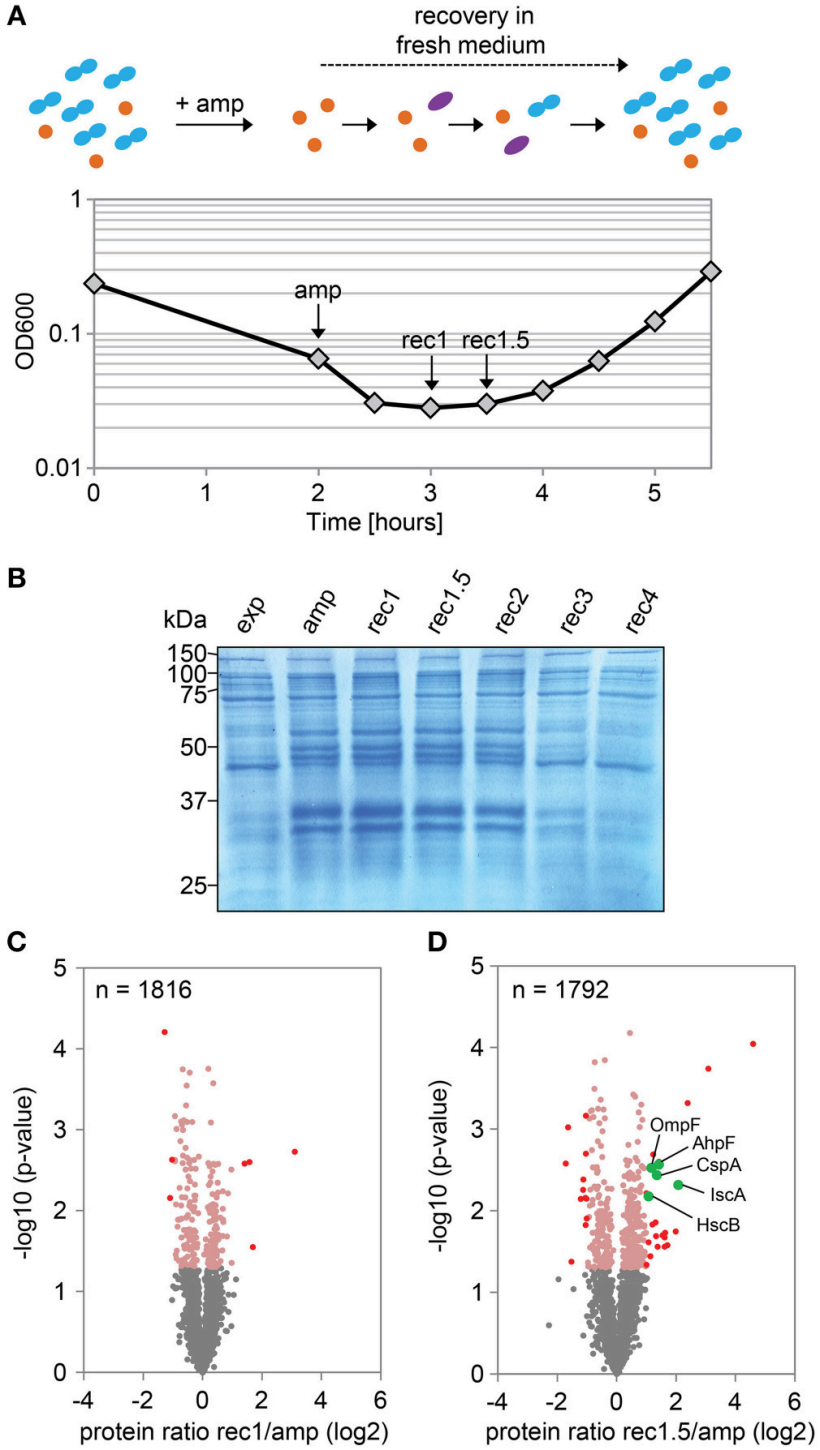
Proteome analysis of persister cells during recovery from ampicillin treatment. **(A)** Graphical illustration of a persister cycle experiment with the highly persistent strain Δ1-41 Δ*istR*. Actively growing cells are blue, persisters are orange, and cells in the process of recovery are purple. Treatment with ampicillin (amp) during exponential growth phase leads to rapid lysis of non-persistent cells. After transfer of surviving persisters into recovery medium, cells eventually awake and resume growth. A representative growth curve is shown and time points for mass spectrometry analysis are indicated. **(B)** SDS-PAGE of protein samples from persister cycle experiments. Proteins (total amount of 50 μg per sample) were separated on 12% polyacrylamide gels and stained with colloidal Coomassie. A size marker (in kDa) is given on the left hand side. **(C,D)** Volcano plots for the comparison between recovery samples and ampicillin treatment. Data are derived from mass spectrometry analysis. Proteins with a significant change in abundance after 1 h **(B)** and 1.5 h **(C)** of recovery are in red (log_2_ ratios >1 or < −1, *P* < 0.05). Pink dots: log_2_ ratios <1 or >-1, *P* < 0.05. Gray dots: log_2_ ratios <1 or >-1, *P* > 0.05. Exp: exponential phase; amp: 2 h ampicillin treatment; rec1: 1 h recovery; rec1.5: 1.5 h recovery; rec2: 2 h recovery; rec3: 3 h recovery; rec4: 4 h recovery.

In a follow-up experiment, the proteome of strain Δ1-41 Δ*istR* was analyzed during the recovery phase. When liquid cultures were treated with a high dose of ampicillin (200 μg ml^−1^) during exponential phase (OD_600_ ~0.3) in LB medium, the optical density clearly dropped within 2 h to values below 0.1 due to lysis of non-persistent cells (Figure 2A). After ampicillin treatment, persister cells were centrifuged and washed to remove cell debris, and finally transferred to fresh medium without antibiotics to enable recovery. After ~2 h of recovery, a slight increase of the optical density was visible, and from there cultures resumed exponential growth to complete a persister cycle (Figure 2A). Protein samples from persister cycle experiments were analyzed by SDS-PAGE, revealing a distinctive change in the protein pattern upon ampicillin treatment compared to exponential phase (Figure 2B). The protein pattern remained unchanged until 2 h of recovery, but reversed to the exponential phase pattern after 3 h of recovery (Figure 2B). Samples for proteome analysis by mass spectrometry were collected in biological triplicates after 2 h of ampicillin challenge (“amp”) and at early time points during recovery (“rec1” and “rec1.5”; Figures 2A,B). Protein samples were analyzed by LC-MS/MS and quantified using a label-free approach (Cox et al., 2014). The retained “label-free quantification” (LFQ) values represent normalized protein intensities and can be used as a proxy for protein abundance. Reliability of the label-free normalization approach was high, as judged from LFQ intensity distributions (Supplementary Figure 3). LFQ intensities were subsequently used to identify proteins with changed abundances between conditions (log_2_ protein ratios >1 or < -1, *P* < 0.05). We reasoned that proteins, which exhibit differential expression during the early stages of recovery, are potentially aiding the recovery process itself. Therefore, samples from the early recovery phase (“rec1” and “rec1.5”) were compared to samples taken during the ampicillin treatment (“amp”). The proteome pattern after 1 h of recovery was almost identical to the pattern of ampicillin-treated cells, and only seven proteins showed a significant change matching our criteria (Table 2 and Figure 2C). However, after 1.5 h in recovery medium, 24 proteins were significantly increased and 12 proteins were significantly reduced in abundance (Table 2 and Figure 2D). Importantly, all 36 proteins showed the same direction of regulation after 1 h of recovery, albeit changes in abundance were less pronounced (Supplementary Figure 4). LFQ intensities from all samples were applied to principal component analysis (PCA) to reduce complexity of the data for illustration in a two-dimensional plot. The first dimension reveals the progressive separation of recovery samples from ampicillin samples, while the second dimension most likely reflects variation between replicates (Supplementary Figure 5). We conclude that (i) the TisB-dependent and ampicillin-challenged “persister proteome” undergoes minor changes in a gradual manner within the first 1.5 h of postantibiotic recovery, and that (ii) the small subset of 36 proteins might represent functions important to the recovery process.

**Table 2:** Proteins with differential abundance during recovery.

**Protein name**	**Protein ratio (log**_****2****_**)**		***P*****-value**		**Description**
	**rec1/amp**	**rec1.5/amp**	**rec1/amp**	**rec1.5/amp**	
RpmH	3.11	4.59	0.0019	9.07E−05	50S ribosomal protein L34
RpmF	1.58	3.08	0.0025	1.82E−04	50S ribosomal protein L32
RpmG	1.41	2.39	0.0026	4.79E−04	50S ribosomal protein L33
IscA	1.13	2.07	0.0707	0.0048	Iron-binding protein IscA
PrfA	1.70	1.99	0.0284	0.0180	Peptide chain release factor 1
YbjQ	0.81	1.71	0.0594	0.0264	UPF0145 protein YbjQ
SmrA	0.97	1.63	0.2685	0.0186	Probable DNA endonuclease SmrA
RlmJ	0.88	1.61	0.1092	0.0212	Ribosomal RNA large subunit methyltransferase J
QueD	1.05	1.61	0.1105	0.0276	6-carboxy-5,6,7,8-tetrahydropterin synthase
QueA	0.45	1.55	0.0302	0.0201	S-adenosylmethionine:tRNA ribosyltransferase-isomerase
AhpF	0.68	1.42	0.0058	0.0027	Alkyl hydroperoxide reductase subunit F
RpsU	0.70	1.42	0.0138	0.0025	30S ribosomal protein S21
SrlA	0.51	1.38	0.4402	0.0277	Glucitol/sorbitol permease IIC component
CspA	0.53	1.35	0.0227	0.0037	Cold shock protein CspA
YniC	0.27	1.33	0.3309	0.0204	2-deoxyglucose-6-phosphate phosphatase
Nth	0.62	1.31	0.1636	0.0139	Endonuclease III
YebC	0.54	1.23	0.0200	0.0020	Probable transcriptional regulatory protein YebC
RibB	−0.09	1.20	0.7793	0.0148	3,4-dihydroxy-2-butanone 4-phosphate synthase
OmpF	0.97	1.17	0.0031	0.0030	Outer membrane protein F
PurM	0.12	1.13	0.8329	0.0361	Phosphoribosylformylglycinamidine cyclo-ligase
MurB	0.16	1.07	0.6072	0.0244	UDP-N-acetylenolpyruvoylglucosamine reductase
HscB	0.62	1.07	0.0517	0.0067	Co-chaperone protein HscB
MetJ	0.31	1.00	0.3302	0.0061	Met repressor
PtsN	0.61	1.00	0.1086	0.0462	Nitrogen regulatory protein
GlnP	−0.78	−1.01	0.0130	0.0125	Glutamine transport system permease protein GlnP
YebF	−1.02	−1.01	0.0024	0.0071	Protein YebF
Ivy	−0.93	−1.03	6.78E−04	6.86E−04	Inhibitor of vertebrate lysozyme
SeqA	−1.28	−1.04	6.22E−05	0.0020	Negative modulator of initiation of replication
NuoK	−0.13	−1.04	0.6921	0.0150	NADH-quinone oxidoreductase subunit K
LivJ	−0.64	−1.05	0.0745	0.0069	Leu/Ile/Val-binding protein
RraA	−0.43	−1.12	0.1030	0.0042	Regulator of ribonuclease activity A
MalM	−0.90	−1.13	0.0084	0.0055	Maltose operon periplasmic protein
RcnB	−0.57	−1.21	0.0516	0.0072	Nickel/cobalt homeostasis protein RcnB
FxsA	−0.44	−1.52	0.0231	0.0423	UPF0716 protein FxsA
YmgD	–	−1.64	–	0.0010	Uncharacterized protein YmgD
CsrD	−0.88	−1.71	0.0392	0.0026	RNase E specificity factor CsrD

### Selection of Proteins From the Recovery Phase for Further Analysis

We selected five candidates from the 24 proteins, which were increased in abundance during recovery (Figure 2D). The pulsed-SILAC approach partly guided the selection process: increased synthesis of catalase KatE during ampicillin treatment (Figure 1F) was indicative of oxidative stress caused by hydrogen peroxide, and increased synthesis of iron storage proteins FtnA, Bfr, and Dps (Figure 1F) implied that excess intracellular iron might have originated from decomposition of iron-sulfur clusters (Fe/S). We therefore selected AhpF, a component of the alkyl hydroperoxide reductase involved in peroxide detoxification, and two proteins with functions in Fe/S assembly, HscB and IscA. The transcriptional activator CspA was selected because its mRNA levels are highest immediately before cell division starts (Brandi et al., 2016), implying an important function for growth resumption. Finally, the outer membrane porin OmpF was selected because it plays a major role for influx of nutrients, antibiotics, and other small compounds (Nikaido, 2003; Pagès et al., 2008).

### AhpF and OmpF Specifically Affect the Colony Appearance Time After Antibiotic Treatment

**Figure 3:**
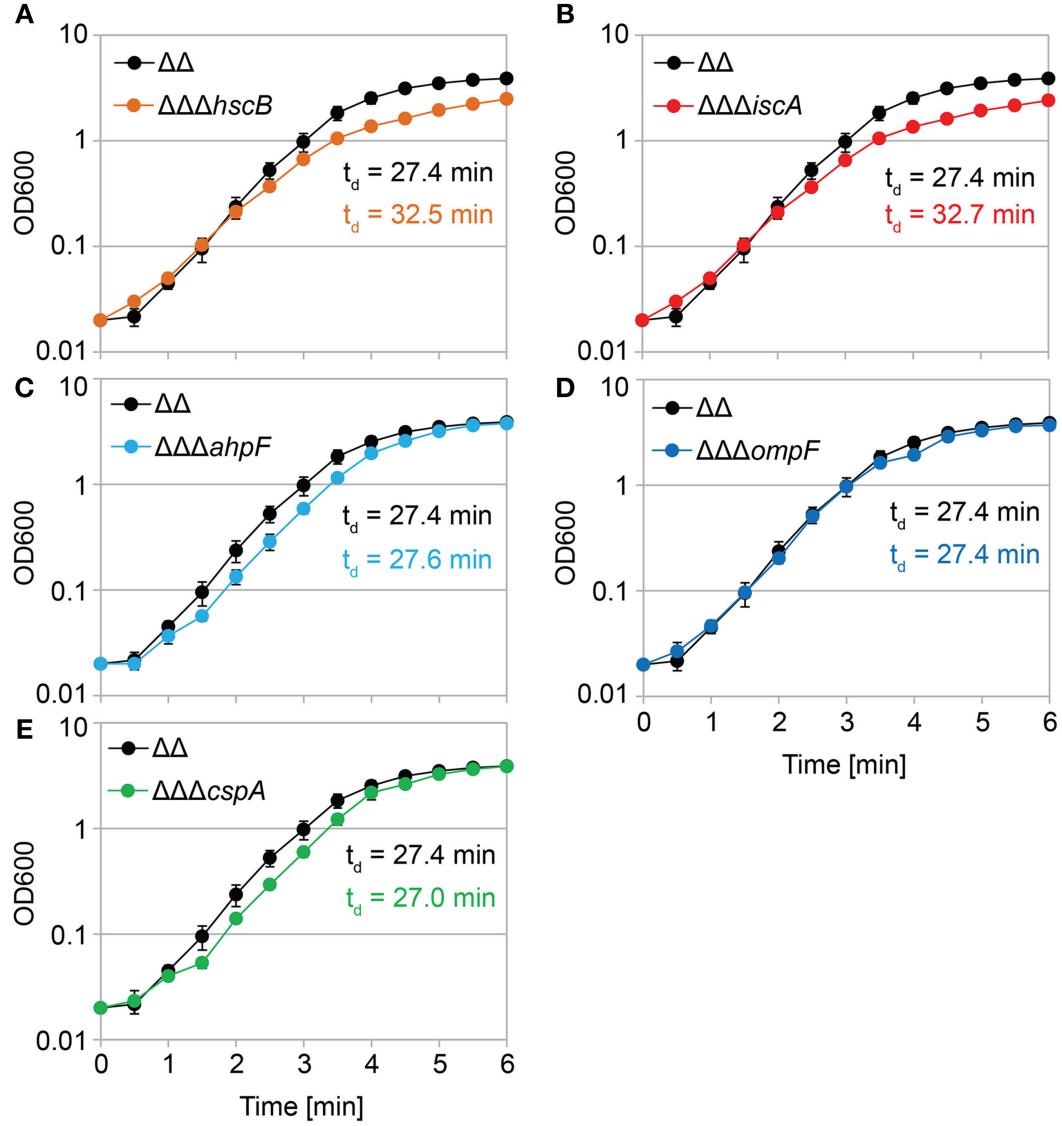
Growth in liquid medium is impaired by *hscB* and *iscA* deletions. Growth curves of double deletion strain Δ1-41 Δ*istR* (ΔΔ) and corresponding triple deletion strains in liquid LB medium. The OD_600_ was initially adjusted to 0.02 from over-night cultures and measured over 6 h. Doubling times (t_d_) during exponential phase are given for the corresponding strains in each graph. **(A)** ΔΔ vs. ΔΔΔ*hscB*, **(B)** ΔΔ vs. ΔΔΔ*iscA*, **(C)** ΔΔ vs. ΔΔΔahpF, **(D)** ΔΔ vs. ΔΔΔ*ompF*, and **(E)** ΔΔ vs. ΔΔΔ*cspA*. Data represent the mean of at least three independent biological replicates. Error bars depict standard deviations.

**Figure 4:**
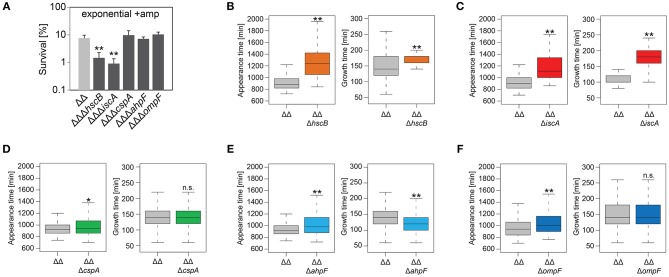
Influence of candidate proteins on survival and recovery of TisB-dependent persister cells after ampicillin treatment. Exponential cultures of double deletion strain Δ1-41 Δ*istR* (ΔΔ) and corresponding triple deletion strains were treated for 3 h with ampicillin (+amp; 200 μg ml^−1^) in liquid LB medium and subsequently plated on LB agar without antibiotics. **(A)** CFU counts before and after ampicillin treatment were used to calculate survival. Data represent the mean of at least three independent biological replicates. Error bars depict standard deviations. *P*-values were calculated using Student's *t*-test (**P < 0.01). **(B–F)** The ScanLag method (Levin-Reisman et al., 2010, 2014) was applied to monitor appearance and growth times of individual colonies after ampicillin treatment. Left and right panels show boxplots for appearance and growth times, respectively, for **(B)** ΔΔ (*n* = 294) vs. ΔΔΔ*hscB* (*n* = 139), **(C)** ΔΔ (*n* = 191) vs. ΔΔΔ*iscA* (*n* = 102), **(D)** ΔΔ (*n* = 190) vs. ΔΔΔ*cspA* (*n* = 203), **(E)** ΔΔ (*n* = 190) vs. ΔΔΔ*ahpF* (*n* = 194), and **(F)** ΔΔ (*n* = 399) vs. ΔΔΔ*ompF* (*n* = 604). *P*-values were calculated using Mann–Whitney-Wilcoxon test (**P < 0.01; *P < 0.05; n.s., not significant).

We constructed gene deletions for *hscB, iscA, cspA, ahpF*, and *ompF* in our highly persistent strain Δ1-41 Δ*istR*. The resulting triple deletion strains were tested with regard to growth and persistence. The doubling time of strain Δ1-41 Δ*istR* was 27.4 min during exponential phase in liquid LB medium. Deletion of *hscB* and *iscA* resulted in significantly increased doubling times of 32.5 and 32.7 min, respectively (Table 3 and Figures 3A,B). In addition, the optical density after 6 h of growth was lower in both strains (Figures 3A,B). We also determined the fraction of surviving cells after 3 h of ampicillin treatment (200 μg ml^−1^) during exponential phase, to test whether persister levels were affected in the triple deletion strains. The persister level of strain Δ1-41 Δ*istR* was determined as 7.5%. Deletion of *hscB* and *iscA* resulted in significantly decreased persister levels of 1.5 and 0.9%, respectively (Figure 4A). By contrast, doubling times and persister levels for the remaining deletion strains (*cspA, ahpF*, and *ompF*) were largely unaffected (Table 3 and Figures 3C–E, 4A). We next asked the question whether the five genes have an effect on the time individual cells remain in the persistent state after an antibiotic challenge. Liquid cultures of the deletion strains were treated with ampicillin (200 μg ml^−1^) for 3 h and subsequently plated on LB agar without antibiotics to enable recovery of the surviving persister cells and formation of colonies. Colony growth was analyzed by the ScanLag method, which has been developed to simultaneously measure the appearance and growth times of hundreds of colonies on agar plates (Levin-Reisman et al., 2010, 2014). The appearance time indicates the very first detection event of a colony, which is represented by a colony size of 10 pixels in the scanned images. The growth time reflects an increase in colony size from 80 to 160 pixels (see section Materials and Methods). If the growth time of a colony is largely unaffected, the appearance time mainly depends on the time the colony-forming cell needs to recover and, therefore, reflects the persistence time. The median appearance time of strain Δ1-41 Δ*istR* ranged between 880 and 940 min and the median growth time was mostly 140 min (Table 4). Deletion of *hscB* and *iscA* resulted in a significant shift to later appearance and growth times, with colonies appearing on average after more than 1,100 min with a growth time of 180 min (Table 4 and Figures 4B,C). These findings validated a general growth defect for the *hscB* and *iscA* deletions, which was apparent both on agar plates and in liquid medium (Tables 3, 4). By contrast, and consistent with measurements in liquid LB medium, deletions of *cspA, ahpF*, and *ompF* did not extend the growth time of colonies (Figures 4D–F). The *ahpF* deletion even had a reduced growth time of 120 min (Table 4). However, the appearance time was shifted to later time points in all three deletion strains, which was particularly evident for *ahpF* and *ompF* deletions. Both strains lost a major fraction of colonies with an early appearance time and gained colonies with an appearance time later than 1,400 min (Figures 4E,F). Since growth time was not affected, the later appearance time indicated a delay in outgrowth of persister cells.

**Table 3:** Doubling times of deletion strains in liquid medium.

**Strain**	**Doubling time [min] Mean ± SD**	***P*-value**
ΔΔ	27.4 ± 0.7	
ΔΔΔ*hscB*	32.5 ± 0.1	1.86E-06
ΔΔΔ*iscA*	32.7 ± 0.9	1.35E-05
ΔΔΔ*cspA*	27.0 ± 0.2	0.2765
ΔΔΔ*ahpF*	27.6 ± 0.1	0.6727
ΔΔΔ*ompF*	27.4 ± 0.3	0.9441

*Doubling times were calculated from exponential growth phase in liquid LB medium (Figure 3). Values represent the mean and standard deviation (SD) of at least three independent biological replicates. Statistical testing using Student's t-test (P-value) refers to double deletion strain Δ1-41 ΔistR (ΔΔ)*.

**Table 4:** Growth parameters of deletion strains on solid medium after antibiotic treatment.

**Condition**	**Appearance time [min]**					**Growth time [min]**			
	**Control strain**	**Median**	**Deletion strain**	**Median**	***P*-value**	**Control strain**	**Median**	**Deletion strain**	**Median**	***P*-value**
Exponential +amp	ΔΔ	880	ΔΔΔ*hscB*	1,240	<2.2E-16	ΔΔ	140	ΔΔΔ*hscB*	180	3.01E-10
	ΔΔ	900	ΔΔΔ*iscA*	1,110	<2.2E-16	ΔΔ	120	ΔΔΔ*iscA*	180	<2.2E-16
	ΔΔ	920	ΔΔΔ*cspA*	940	0.0363	ΔΔ	140	ΔΔΔ*cspA*	140	0.4227
	ΔΔ	920	ΔΔΔ*ahpF*	980	2.37E-06	ΔΔ	140	ΔΔΔ*ahpF*	120	8.01E-05
	ΔΔ	940	ΔΔΔ*ompF*	1,000	5.75E-10	ΔΔ	140	ΔΔΔ*ompF*	140	0.3795
Exponential +CF	ΔΔ	1,480	ΔΔΔ*hscB*	1,650	4.77E-07	ΔΔ	140	ΔΔΔ*hscB*	200	<2.2E-16
	ΔΔ	1,480	ΔΔΔ*iscA*	1,620	0.0026	ΔΔ	140	ΔΔΔ*iscA*	220	<2.2E-16
	ΔΔ	1,290	ΔΔΔ*ahpF*	1,360	0.0294	ΔΔ	120	ΔΔΔ*ahpF*	120	0.2841
	ΔΔ	1,290	ΔΔΔ*ompF*	1,360	0.0355	ΔΔ	120	ΔΔΔ*ompF*	140	0.0039
Exponential +amp	wt	760	Δ*hscB*	900	<2.2E-16	wt	140	Δ*hscB*	200	<2.2E-16
	wt	760	Δ*iscA*	960	<2.2E-16	wt	140	Δ*iscA*	220	<2.2E-16
	wt	820	Δ*ahpF*	820	0.5294	wt	140	Δ*ahpF*	140	0.1106
	wt	760	Δ*ompF*	760	0.1024	wt	140	Δ*ompF*	140	0.6671
Exponential +amp	Δ*tisB*	820	Δ*tisB* Δ*ahpF*	820	0.2650	Δ*tisB*	160	Δ*tisB* Δ*ahpF*	120	<2.2E-16
	Δ*tisB*	820	Δ*tisB* Δ*ompF*	780	5.25E-13	Δ*tisB*	160	Δ*tisB* Δ*ompF*	140	2.89E-05
Stationary +CF	wt	1,080	Δ*ahpF*	1,080	0.6382	wt	140	Δ*ahpF*	140	0.7855
	wt	1,080	Δ*ompF*	1,030	0.1983	wt	140	Δ*ompF*	120	1.41E-05
Exponential +amp	ΔΔ	960	ΔΔΔ*ahpF*	1,140	8.24E-13	ΔΔ	140	ΔΔΔ*ahpF*	140	0.0017
	ΔΔ	960	ΔΔΔ*ahpF* Δ*ompF*	1,000	0.0004	ΔΔ	140	ΔΔΔ*ahpF* Δ*ompF*	120	<2.2E-16

**Figure 5:**
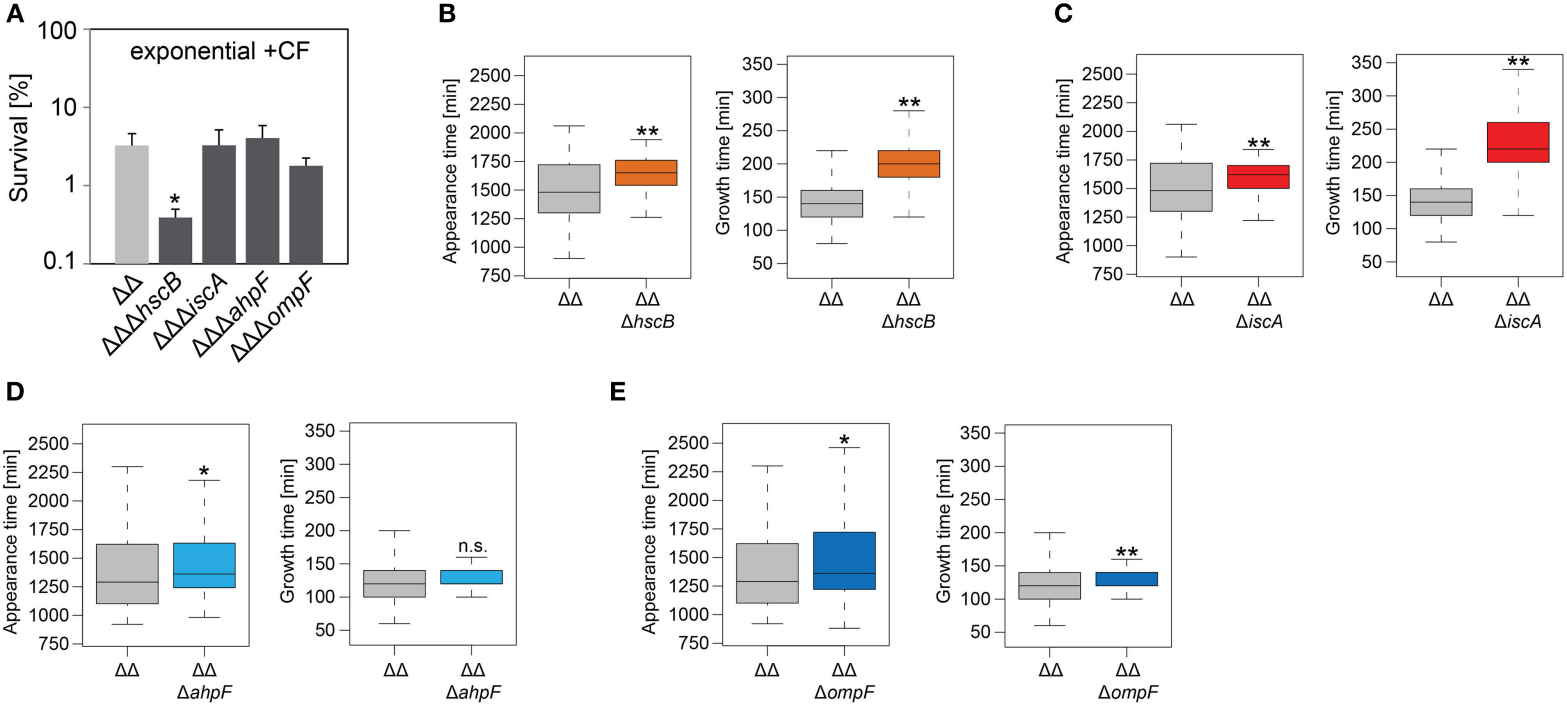
Influence of candidate proteins on survival and recovery of TisB-dependent persister cells after ciprofloxacin treatment. Exponential cultures of double deletion strain Δ1-41 Δ*istR* (ΔΔ) and corresponding triple deletion strains were treated for 3 h with ciprofloxacin (+CF; 10 μg ml^−1^) in liquid LB medium and subsequently plated on LB agar without antibiotics. **(A)** CFU counts before and after ciprofloxacin treatment were used to calculate survival. Data represent the mean of at least three independent biological replicates. Error bars depict standard deviations. *P*-values were calculated using Student's *t*-test (*P < 0.05). **(B–E)** The ScanLag method (Levin-Reisman et al., 2010, 2014) was applied to monitor appearance and growth times of individual colonies after ciprofloxacin treatment. Left and right panels show boxplots for appearance and growth times, respectively, for **(B)** ΔΔ (*n* = 133) vs. ΔΔΔ*hscB* (*n* = 210), **(C)** ΔΔ (*n* = 133) vs. ΔΔΔ*iscA* (*n* = 215), **(D)** ΔΔ (*n* = 118) vs. ΔΔΔ*ahpF* (*n* = 104), and **(E)** ΔΔ (*n* = 118) vs. ΔΔΔ*ompF* (*n* = 75). *P*-values were calculated using Mann–Whitney-Wilcoxon test (**P < 0.01; *P < 0.05; n.s., not significant).

**Figure 6:**
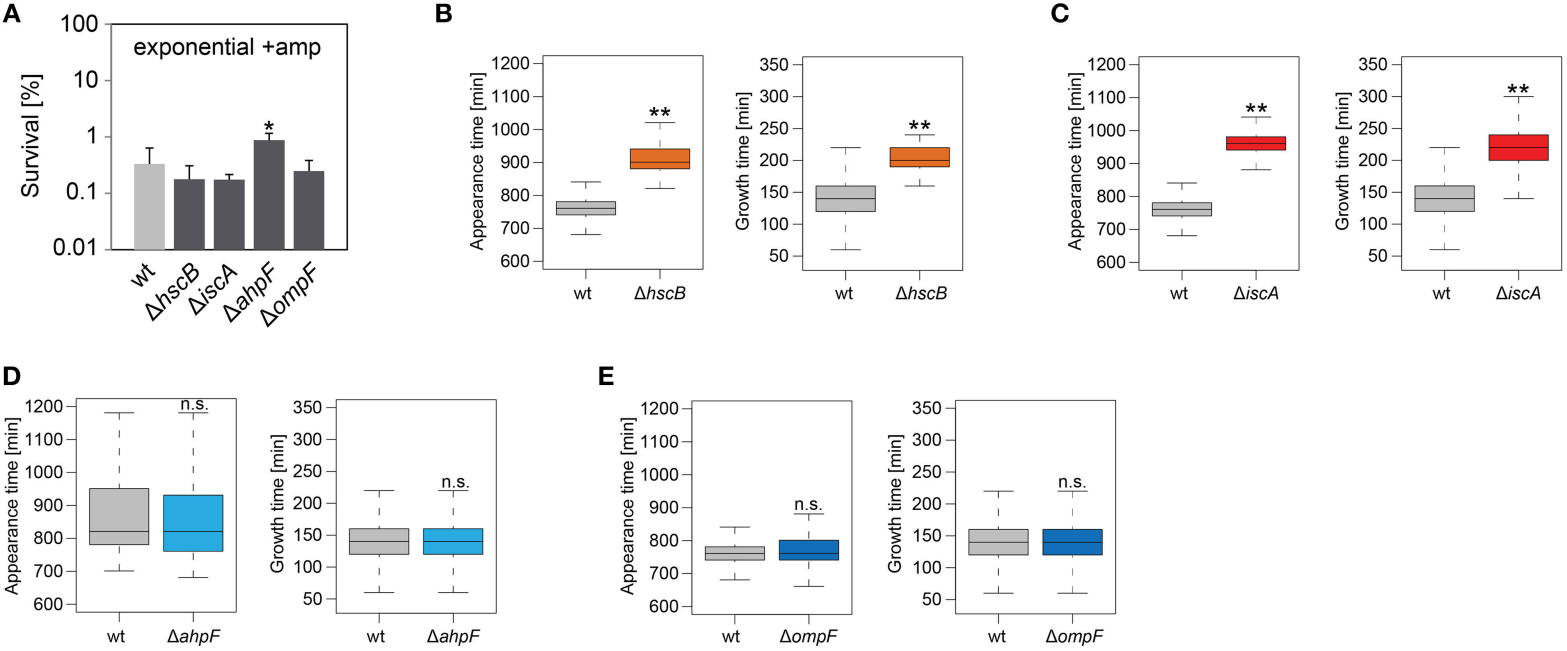
Influence of candidate proteins on survival and recovery of wild-type persister cells after ampicillin treatment. Exponential cultures of wild type MG1655 (wt) and corresponding deletion strains were treated for 3 h with ampicillin (+amp; 200 μg ml^−1^) in liquid LB medium and subsequently plated on LB agar without antibiotics. **(A)** CFU counts before and after ampicillin treatment were used to calculate survival. Data represent the mean of at least three independent biological replicates. Error bars depict standard deviations. *P*-values were calculated using Student's *t*-test (*P < 0.05). **(B–E)** The ScanLag method (Levin-Reisman et al., 2010, 2014) was applied to monitor appearance and growth times of individual colonies after ampicillin treatment. Left and right panels show boxplots for appearance and growth times, respectively, for **(B)** wt (*n* = 80) vs. Δ*hscB* (*n* = 84), **(C)** wt (*n* = 80) vs. Δ*iscA* (*n* = 144), **(D)** wt (*n* = 224) vs. Δ*ahpF* (*n* = 219), and **(E)** wt (*n* = 276) vs. Δ*ompF* (*n* = 391). *P*-values were calculated using Mann-Whitney-Wilcoxon test (**P < 0.01; n.s., not significant).

**Figure 7:**
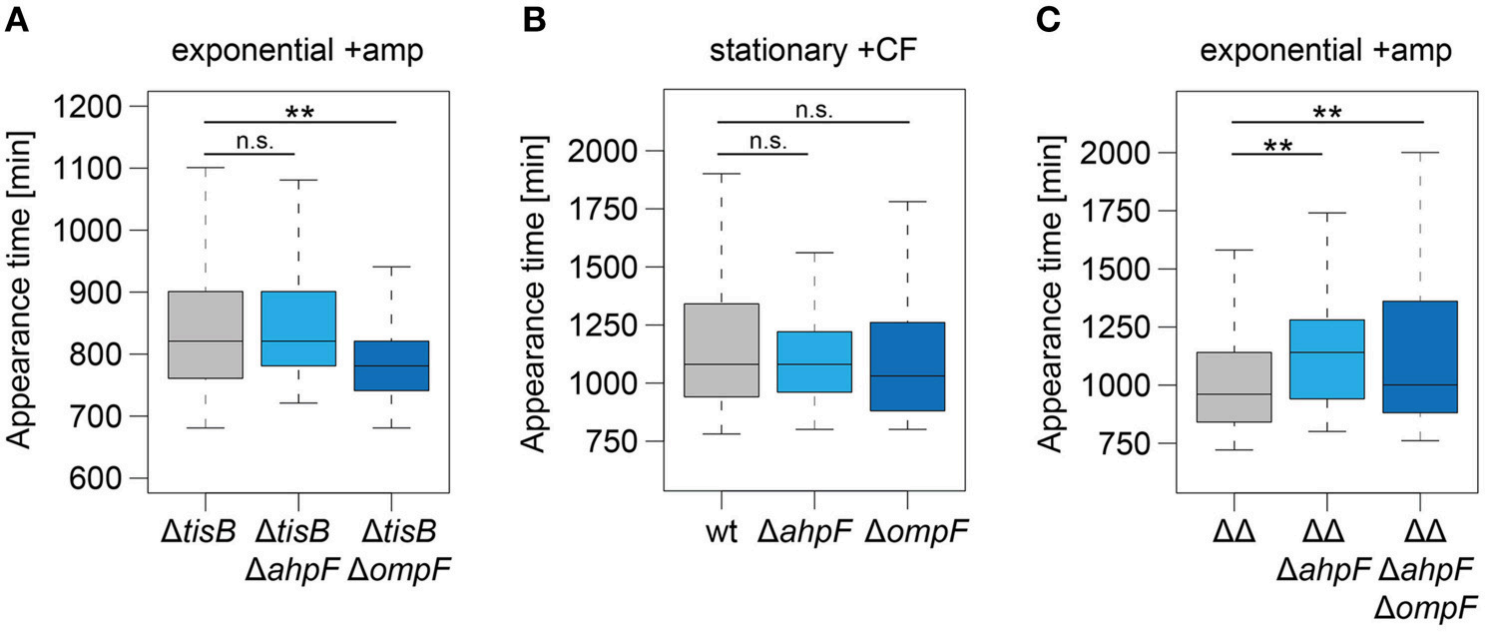
The role of AhpF and OmpF in TisB-independent persisters and synergistic effects. Deletions of *ahpF* and *ompF* were constructed in a *tisB* deletion (Δ*tisB*), wild-type (wt), or Δ1-41 Δ*istR* (ΔΔ) background. Resulting strains were analyzed by ScanLag. **(A)** Cultures were treated with ampicillin (+amp; 200 μg ml^−1^) for 3 h during exponential phase. Boxplots reflect appearance time distributions for Δ*tisB* (*n* = 394), Δ*tisB* Δ*ahpF* (*n* = 204), and Δ*tisB* Δ*ompF* (*n* = 272). **(B)** Cultures were treated with ciprofloxacin (+CF; 10 μg ml^−1^) for 5 h during stationary phase. Boxplots reflect appearance time distributions for wt (*n* = 113), Δ*ahpF* (*n* = 147), and Δ*ompF* (*n* = 58). **(C)** Cultures were treated with ampicillin (+amp; 200 μg ml^−1^) for 3 h during exponential phase. Boxplots reflect appearance time distributions for ΔΔ (*n* = 325), ΔΔΔ*ahpF* (*n* = 233), and ΔΔΔ*ahpF*Δ*ompF* (*n* = 142). *P*-values were calculated using Mann–Whitney-Wilcoxon test (**P < 0.01; n.s., not significant).

*Appearance and growth times represent median values of ScanLag experiments (Figures 4–7). Statistical testing using Mann–Whitney-Wilcoxon test (P-value) refers to the corresponding control strain from the same experimental run*.

We repeated the experiment with the fluoroquinolone antibiotic ciprofloxacin and treated exponentially growing cultures with a high dose (10 μg ml^−1^) for 3 h. The *cspA* deletion was not further investigated, since it has only caused a small effect on the colony appearance time after treatment with ampicillin. The persister level of strain Δ1-41 Δ*istR* was determined as 3.2% for ciprofloxacin, and a significant decrease to 0.4% was only observed for the *hscB* deletion (Figure 5A). After ciprofloxacin treatment, all strains had a median growth time on LB agar which was comparable to growth times obtained after ampicillin treatment. However, the appearance time distributions after ciprofloxacin treatment were overall shifted to later time points, when individual strains were compared to the respective ampicillin experiment (Table 4; compare Figures 4, 5). In case of ciprofloxacin, deletion of *hscB* and *iscA* caused a shift of ≥140 min for the median appearance time and ≥60 min for the median growth time in comparison to strain Δ1-41 Δ*istR* (Table 4 and Figures 5B,C). By contrast, *ahpF* and *ompF* deletions caused a delay in colony appearance (70-min shift of the median appearance time), without severely affecting the growth time (Table 4 and Figures 5D,E).

As a conclusion, the growth phenotypes obtained for the *hscB, iscA, ahpF*, and *ompF* deletions were not specific to a distinct antibiotic, but instead applied to at least two different classes of antibiotics (β-lactams and fluoroquinolones). Furthermore, deletion of *ahpF* and *ompF* specifically affected the colony appearance time after antibiotic treatment in strain Δ1-41 Δ*istR*, possibly due to impairment of the recovery process.

### Functions of AhpF and OmpF During Recovery Are Specific to TisB-Dependent Persister Cells

We were curious whether our findings were influenced by the genetic background of strain Δ1-41 Δ*istR*. To answer this question, *hscB, iscA, ahpF*, and *ompF* deletions were constructed in the MG1655 wild-type background. Strains were treated with ampicillin (200 μg ml^−1^) for 3 h during exponential phase, as before, and analyzed by ScanLag. Interestingly, none of the deletions caused a reduction in persister levels (Figure 6A). While *hscB* and *iscA* deletions caused a shift to later appearance and growth times compared to wild type MG1655 (Table 4 and Figures 6B,C), growth parameters of *ahpF* and *ompF* deletion strains were almost identical to those obtained for the wild type (Table 4 and Figures 6D,E). The influence of *ahpF* and *ompF* on the colony appearance time (after ampicillin treatment) was therefore no general feature and appeared to be specific for TisB-dependent persister cells.

To further corroborate our findings, *ahpF* and *ompF* deletions were constructed in a Δ*tisB* background and analyzed as before. Deletion of *ahpF* did not affect the persistence time, while deletion of *ompF* even caused an earlier appearance of colonies (Table 4 and Figure 7A). In a next experiment, wild-type *ahpF* and *ompF* deletion strains were treated with ciprofloxacin (10 μg ml^−1^) during stationary phase for 5 h. ScanLag analysis revealed that the colony appearance time of stationary phase persisters was neither affected by *ahpF* nor *ompF* deletions (Table 4 and Figure 7B). These experiments confirmed that AhpF and OmpF specifically affect recovery of TisB-dependent persister cells, but excluded a more general role in recovery of persister cells that have formed through other mechanisms.

### Simultaneous Deletion of a*hpF* and o*mpF* Does not Cause a Synergistic Effect

Since ScanLag experiments with *ahpF* and *ompF* single deletions in strain Δ1-41 Δ*istR* indicated that both genes support the recovery process of TisB-dependent persister cells (Figures 4, 5), we asked the question whether simultaneous deletion of both genes would cause a severe delay in colony appearance. To this end, a marker-less variant of strain Δ1-41 Δ*istR* was constructed by FLP-mediated recombination. Subsequent deletion of *ahpF*, resulting in a triple deletion strain, caused a prolonged appearance time of colonies after ampicillin treatment during exponential phase (Figure 7C), which confirmed our former findings (Figure 4E). Additional deletion of *ompF* produced a quadruple deletion strain, which was still highly persistent (Supplementary Figure 6). However, the colony appearance time in the quadruple deletion strain was not further increased in comparison to the triple deletion strain (Figure 7C). On the contrary, the median appearance time of the quadruple deletion strain was even intermediate between the initial strain (Δ1-41 Δ*istR*) and the triple deletion strain.

## Discussion

Antibiotic-tolerant persister cells increase the risk for relapsing infections, a threat especially prevalent in combination with bacterial biofilms (Lewis, 2007, 2010; Michiels et al., 2016). Assessing persister physiology is key to understanding the processes that drive bacterial persistence, which will ultimately guide development of therapeutic strategies. Several studies have assessed the persister transcriptome by microarray or RNA-seq analysis after enrichment of persister fractions by either lysis of non-persistent cells (Keren et al., 2004, 2011; Pu et al., 2016), or fluorescence-activated cell sorting (FACS) of reporter strains (Shah et al., 2006). While each of these enrichment methods has its limitations in terms of purity of the enriched persister fractions (Cañas-Duarte et al., 2014; Henry and Brynildsen, 2016), the transcriptome data provided conclusive insights into persister physiology. For example, pioneering work from the Lewis group highlighted the prevalence of toxin mRNAs in persister fractions and emphasized the importance of chromosomally encoded TA systems for bacterial persistence (Keren et al., 2004; Shah et al., 2006). However, alterations on transcript level do not necessarily affect the amount of a given protein due to posttranscriptional and posttranslational regulatory events. To complement the picture of persister physiology, high-throughput investigations of the proteome are needed, but have rarely been addressed.

### Pulsed-SILAC Reveals Persister Physiology on the Translational Level

Pulsed-SILAC is a powerful tool to globally assess active protein translation (Schwanhäusser et al., 2009), but even though it has been successfully applied to study, e.g., protein synthesis in colistin-tolerant subpopulations of *Pseudomonas aeruginosa* biofilms (Chua et al., 2016), it has not yet been applied to planktonic persister cells during an antibiotic challenge. Here, a pulsed-SILAC approach was used to quantify protein synthesis in TisB-dependent persister cells after an ampicillin challenge during exponential phase (Figure 1B). The enriched persister fraction exhibited a >2-fold reduction in protein synthesis compared to the exponentially growing control, as judged from the average Lys8 incorporation of 33.6 and 71.9%, respectively (Figures 1C,D). These findings are in line with the general notion that the likelihood of persister formation inversely correlates with the translational activity (Balaban et al., 2004; Shah et al., 2006; Orman and Brynildsen, 2013; Henry and Brynildsen, 2016). Additionally, Lys8 incorporation might be compromised in TisB-dependent persisters, since depolarization by TisB and subsequent ATP depletion (Unoson and Wagner, 2008; Gurnev et al., 2012) is expected to impede lysine uptake by ABC transporters. Even though protein synthesis is diminished on average, there is high variability in protein synthesis with 43 proteins exhibiting elevated levels of Lys8 incorporation (~45–80%; Figures 1E,F). These proteins likely represent an active stress response in TisB-dependent persister cells. In an alternative and toxin-independent model of persistence, high levels of persister cells are formed after nutrient shifts, e.g., from glucose to fumarate. Quantitative proteomics revealed activation of a distinct stress response, mainly controlled by the sigma factor RpoS (Radzikowski et al., 2016). Interestingly, TisB-dependent persister cells strongly synthesize RpoS (Lys8 incorporation of 77.7%) and the RpoS-dependent proteins Dps, KatE, Cfa, OsmY, OtsA, and PoxB (Figure 1F), most of which serve protective functions. For some of them, enrichment in persister fractions has already been shown on the mRNA level by transcriptome analysis (Keren et al., 2004; Shah et al., 2006), which applies to KatE (catalase II), OsmY (periplasmic chaperone), OtsA (trehalose-6-phosphate synthase), and to the dual-function effector of the envelope stress response PspA. These proteins might represent a general hallmark of persister proteomes and serve as suitable biomarkers for persister cells in future studies. Other proteins found by our pulsed-SILAC approach might influence persistence directly through inhibition of replication (CspD) (Kim and Wood, 2010) or inhibition of translation (RaiA). In summary, we conclude that TisB-dependent persister cells mount an active response on the translational level, which has the potential to (i) actively protect persister cells from severe damage and (ii) induce or stabilize the persistent state. The question to which extent the proteins identified here contribute to TisB-dependent persistence—or persistence in general—needs to be addressed in future studies.

Assuming that the particular response to a stress factor (e.g., antibiotics or nutrient deprivation) depends on the molecular status (e.g., expression of TA systems or metabolic state) of a persister cell, it seems reasonable that the same stress would elicit different responses in different persister types. For example, stationary phase persisters might react in a different way to ciprofloxacin than depolarized persisters generated during exponential phase. Systematic investigations of the persister proteome from a set of defined persister populations might help to unravel shared and specialized features of stress responses in different persister types. Our pulsed-SILAC approach represents a first step toward this direction.

### Postantibiotic Recovery of TisB-Dependent Persister Cells

Mechanisms that lead to persister formation are diverse but in many instances quite well-understood. Mechanisms that help bacteria to recover from the persistent state, however, are only beginning to be discovered, and can be classified as follows: (i) rescuing of targets, that have been corrupted by toxins, to enable awakening (De Jonge et al., 2009; Cheverton et al., 2016), (ii) intrinsic regulatory features of TA operons by a process called “conditional cooperativity” to regain inhibition of toxins by their cognate antitoxins (Page and Peti, 2016), and (iii) repair of antibiotic-induced damages during the postantibiotic recovery phase to maintain survival (Völzing and Brynildsen, 2015; Mok and Brynildsen, 2018). Here, we applied label-free quantitative MS to identify differentially expressed proteins in TisB-dependent persisters during recovery from ampicillin. Importantly, our sampling time points precede bulk growth resumption (Figures 2A,B), which is also reflected by the small proportion of proteins with decreased (12 proteins) or enhanced abundance (24 proteins) after 1.5 h of recovery (Figure 2D). At this time point, TisB persisters have obviously just started to remodel their proteome, and are—from a physiological point of view—still engaged in an intermediate state between persistence and growth resumption.

The proteomics snapshot identified proteins harboring interesting functions with respect to what we have learned from the pulsed-SILAC approach. Increased synthesis of catalase KatE during ampicillin treatment of strain Δ1-41 Δ*istR* (Figure 1F) indicates that TisB persisters need to detoxify hydrogen peroxide. Alternatively, increased KatG synthesis might be an inevitable consequence of activation of the general stress response by RpoS (Figure 1F) without increased ROS production. However, generation of ROS by antibiotics is a documented, albeit controversial, phenomenon (Kohanski et al., 2007; Dwyer et al., 2014), and we have reason to believe that TisB, and other depolarizing toxins, further enhance ROS production (our unpublished data). It is, therefore, feasible to assume that TisB persisters accumulate oxidative damage that needs to be repaired during postantibiotic recovery. In this regard, TisB-dependent persisters might be reminiscent of viable but non-culturable (VBNC) *E. coli* cells, that exhibit signatures of oxidative protein damage and have activated RpoS-dependent genes like *katE* (Desnues et al., 2003). But unlike VBNC cells, TisB persisters are able to produce colonies in a timely fashion, even though their appearance time is delayed due to high TisB levels (compare wt and ΔΔ in Table 4) (Berghoff et al., 2017). We observed increasing protein levels of AhpF, a component of alkyl hydroperoxide reductase Ahp, during recovery of strain Δ1-41 Δ*istR* (Figure 2D). Ahp is the primary scavenger of hydrogen peroxide under standard growth conditions (Imlay, 2013). Furthermore, Ahp has the potential to reduce a variety of alkyl hydroperoxides (Jacobson et al., 1989; Storz et al., 1989), and might be involved in repairing damaged molecules in TisB-dependent persister cells during postantibiotic recovery. In line with this hypothesis, the colony appearance time after antibiotic treatment was prolonged in strain Δ1-41 Δ*istR* when *ahpF* was deleted (Figures 4E, 5D). Since persister levels were not affected (Figures 4A, 5A), the amount of oxidative damage in TisB persisters appears to be sublethal, maybe due to hydrogen peroxide detoxification by catalase KatE already during the persistent state. Interestingly, the effect of an *ahpF* deletion on the appearance time was not observed in a wild-type or Δ*tisB* background (Figures 6D, 7A). Furthermore, an *ahpF* deletion did not extend the lag phase after diluting cells from stationary phase into fresh LB medium (Figure 3C). Together these data demonstrate that AhpF does not represent a crucial factor during outgrowth of *E. coli* in general. The specific importance of AhpF for recovery of TisB persisters rather supports the assumption that oxidative stress represents a particular threat for depolarized cells.

The pulsed-SILAC approach also demonstrated increased synthesis of iron storage proteins FtnA, Bfr, and Dps (Figure 1F), which was indicative of excess free iron within TisB persisters. Free iron might originate from decomposition of Fe/S by ROS (Imlay, 2006, 2008), and iron sequestration is, therefore, needed to avoid subsequent generation of genotoxic hydroxyl radicals through Fenton chemistry. In line with the proposed Fe/S decomposition, proteins with a function in Fe/S assembly were upregulated during recovery, which applies to the A-type Fe/S carrier protein IscA and chaperone HscB, which is involved in release of Fe/S from scaffold proteins (Roche et al., 2013). Growth defects have been reported for both *hscB* and *iscA* deletion strains (Tokumoto and Takahashi, 2001; Lu et al., 2008), which was also observed here in the Δ1-41 Δ*istR* background (Table 3) and for wild type MG1655 (data not shown). The general growth defect likely causes the strongly delayed appearance time of colonies in ScanLag experiments (Figures 4–6), and it is, therefore, difficult to draw conclusions about duration of the persistent state from these colony-based experiments. However, upon ampicillin treatment persister levels of strain Δ1-41 Δ*istR* were reduced by ~5- and 8-fold due to *hscB* and *iscA* deletions, respectively (Figure 4A), which was not observed in a wild-type background (Figure 6A). We speculated that the reduced survival was caused by generation of hydroxyl radicals, which could not be confirmed in experiments with the hydroxyl radical scavenger thiourea (data not shown). We conclude that Fe/S assembly specifically supports the postantibiotic recovery process of TisB persisters to maintain survival, but cannot exclude an additional role in persister generation.

### The Ability of OmpF to Influence Recovery Depends on the Physiological Condition

The intracellular concentration of an antibiotic is determined by two processes: influx and efflux. Efflux of antibiotics by bacterial pumps is mainly considered an important determinant for antibiotic resistance. It was only recently shown that *E. coli* persister cells extrude β-lactams by TolC-dependent pumps as an active defense and survival strategy (Pu et al., 2016). However, the parallel induction of *ompF*, as monitored on RNA level, was considered a paradox, since OmpF is a major entry gate for β-lactams and other antibiotics (Nikaido, 2003; Pagès et al., 2008). These results were interpreted as lack of cooperation between efflux and influx systems (Pu et al., 2016). Our data might provide a solution to this problem. One has to consider that OmpF and other porins do not only allow antibiotics to enter the periplasm, but also provide the nutrient supply that is needed for cell growth. If *ompF* is deleted in a Δ*tisB* background, cells recover more quickly after an ampicillin challenge (Figure 7A), likely because intracellular ampicillin accumulation is reduced. The situation reverses in TisB persisters: deleting *ompF* in the Δ1-41 Δ*istR* background causes a prolonged persistence time (Figures 4F, 5E), indicating that OmpF is an important factor for recovery. Some nutrients, like sugars and other metabolites, have the potential to repolarize the inner membrane and reverse toxin-dependent depolarization (Allison et al., 2011; Verstraeten et al., 2015). Increased nutrient supply by OmpF upregulation might support this process. We conclude that high levels of OmpF are either detrimental or beneficial for persister cells, depending on the particular persistence mechanism and physiological condition. The surprising lack of synergistic effects in the quadruple deletion strain (Δ1-41 Δ*istR* Δ*ahpF* Δ*ompF*, Figure 7C) can be explained along the same lines. If AhpF is not present in TisB persisters, the physiological condition has changed. Now, an *ompF* deletion turns out to be beneficial for the recovery process. In summary, the functional importance of a particular protein during postantibiotic recovery of persister cells strongly depends on the physiological condition and is expected to show high variations on the single cell level among mixed persister populations.

## Conclusions

Proteome analysis by state-of-the-art MS is a powerful tool to assess persister physiology, and was applied here to learn more about proteins with potential functions during postantibiotic recovery. TisB-dependent persisters were chosen as a model system for “persistence by depolarization.” The investigated proteins with increased abundance during recovery fall into three classes: (i) proteins with no major impact, neither on persister level nor persistence time (CspA), (ii) proteins important for growth in general and persister survival in particular (HscB and IscA), and (iii) proteins with specific functions during recovery of TisB persisters (AhpF and OmpF). How expression of these proteins is regulated during the recovery phase remains an exciting question for future studies.

## Data Availability

All datasets generated or analyzed for this study are included in the manuscript and/or the supplementary files.

## Author Contributions

AK and BB designed the study. D-TS performed most of the physiological experiments and established a workflow for analysis of ScanLag data. DE and BB performed additional physiological experiments and analyzed the data. AK performed mass spectrometry, analyzed the data, and contributed to the material and methods section. BB wrote the manuscript. All authors read and approved the final manuscript.

### Conflict of Interest Statement

The authors declare that the research was conducted in the absence of any commercial or financial relationships that could be construed as a potential conflict of interest.
