# Supplementary material for: High-Throughput Proteomics Identifies Proteins With Importance to Postantibiotic Recovery in Depolarized Persister Cells
Source: Front Microbiol. 2019 Mar 6;10:378. doi: 10.3389/fmicb.2019.00378 (PMC6414554; doi:10.3389/fmicb.2019.00378)
Supplement: Supplementary file 1 [file Table_1.DOCX]

**Supplementary Table 1. Strains and plasmids used in this study.**

**Strain Relevant features Source/Reference**

MG1655 K-12 F^–^ λ^–^

Δ*tisAB*::FRT Markerless deletion of *tisAB* Dörr et al., 2010

B133 Scarless deletion of +1-41 region of *tisB* 5'-UTR and Berghoff et al., 2017

deletion of *istR* (Δ1-41 Δ*istR::frt-kan-frt*), Km^R^

DTS-5 Deletion of *ahpF* with *cat* (Δ*ahpF::cat*) in MG1655, Cm^R^ This study

DTS-7 Deletion of *ompF* with *cat* (Δ*ompF::cat*) in MG1655, Cm^R^ This study

DE-73 Deletion of *iscA* with *cat* (Δ*iscA::cat*) in MG1655, Cm^R^ This study

DE-75 Deletion of *hscB* with *cat* (Δ*hscB::cat*) in MG1655, Cm^R^ This study

DE-38 Deletion of *ahpF* with *cat* (Δ*ahpF::cat*) in Δ*tisAB*::FRT, Cm^R^ This study

DE-39 Deletion of *ompF* with *cat* (Δ*ompF::cat*) in Δ*tisAB*::FRT, Cm^R^ This study

DTS-8 Deletion of *ahpF* with *cat* (Δ*ahpF::cat*) in B133, Km^R^, Cm^R^ This study

DTS-9 Deletion of *cspA* with *cat* (Δ*cspA::cat*) in B133, Km^R^, Cm^R^ This study

DTS-10 Deletion of *ompF* with *cat* (Δ*ompF::cat*) in B133, Km^R^, Cm^R^ This study

DTS-15 Deletion of *hscB* with *cat* (Δ*hscB::cat*) in B133, Km^R^, Cm^R^ This study

DTS-18 Deletion of *iscA* with *cat* (Δ*iscA::cat*) in B133, Km^R^, Cm^R^ This study

GC-59 Elimination of *kan* in B133 by FLP-mediated recombination This study

(Δ1-41 Δ*istR*::FRT)

GC-67 Deletion of *ahpF* with *cat* (Δ*ahpF::cat*) in GC-59, Cm^R^ This study

GC-68 Deletion of *ompF* with *kan* (Δ*ompF::kan*) in GC-67, Cm^R^, Km^R^ This study

**Plasmid Relevant features Source/Reference**

pSIM5 λ red expression vector, pSC101 *ori*, *repA^ts^*, Tet^R^ Datta et al., 2006

709-FLPe FLPe expression plasmid, pSC101-ts *ori*, Amp^R^ Gene Bridges

**Supplementary References**

Berghoff, B. A., Hoekzema, M., Aulbach, L., and Wagner, E. G. H. (2017). Two regulatory RNA elements affect TisB-dependent depolarization and persister formation. Mol. Microbiol. 103, 1020–1033. doi:10.1111/mmi.13607.

Datta, S., Costantino, N., and Court, D. L. (2006). A set of recombineering plasmids for gram-negative bacteria. Gene 379, 109–115. doi:10.1016/j.gene.2006.04.018.

Dörr, T., Vulic, M., and Lewis, K. (2010). Ciprofloxacin causes persister formation by inducing the TisB toxin in *Escherichia coli*. PLoS Biol 8, e1000317. doi:10.1371/journal.pbio.1000317.
