# Supplementary material for: High-Throughput Proteomics Identifies Proteins With Importance to Postantibiotic Recovery in Depolarized Persister Cells
Source: Front Microbiol. 2019 Mar 6;10:378. doi: 10.3389/fmicb.2019.00378 (PMC6414554; doi:10.3389/fmicb.2019.00378)
Supplement: Supplementary file 2 [file Table_2.DOCX]

**Supplementary Table 2. Oligodeoxyribonucleotides used in this study.**

**Name Sequence (5’ to 3’) Description**

hscB-KO-1 ATTCGCCGTTATCGCGGCGGATCGCAGCCCTGAGAAT Deletion of *hscB* with *cat*

GTTGCTCATATGAATATCCTCCTTAG

hscB-KO-2 TTCACTAATTTGTAATAAGGCCATGTTTAGCTTCCAG Deletion of *hscB* with *cat*

AAACTAGAGCTAACTAACTTGTAGGCTG

hscB-scr-1 CACCGTGGTCGCCTGC Screening of *hscB* deletion

hscB-scr-2 CGCTGATGCGGCGCAG Screening of *hscB* deletion

cspA-KO-1 ATCGCCGAAAGGCACACTTAATTATTAAAGGTAATAC Deletion of *cspA* with *cat*

ACTGCTCATATGAATATCCTCCTTAG

cspA-KO-2 AAATGGCAGGGATCTTAGATTCTGTGCTTTTAAGCAG Deletion of *cspA* with *cat*

AGAGCCTTTGAGTGAGCTGATAC

cspA-scr-1 CCTCGTAGTGCACATTCC Screening of *cspA* deletion

cspA-scr-2 CCAAATGGCAGGGATC Screening of *cspA* deletion

ahpF-KO-1 GGCCGCTTGCATGATGATGTTTAAAGCCCAGGAGATA Deletion of *ahpF* with *cat*

AACGCTCATATGAATATCCTCCTTAG

ahpF-KO-2 CCCCGGCGGCTAAGCAATTGCAGGTGAATCTTACTTCT Deletion of *ahpF* with *cat*

TCGCCTTTGAGTGAGCTGATAC

ahpF-scr-1 CCGCACCATGATGCAAG Screening of *ahpF* deletion

ahpF-scr-2 CCTGCCCGGAGCCATC Screening of *ahpF* deletion

iscA-KO-1 AAAAGCAAACGTGAAGCAAAATAAGAGTTGAGGTTTG Deletion of *iscA* with *cat*

GTTGCTCATATGAATATCCTCCTTAG

iscA-KO-2 CCACGCGCAGGCGACCACGGTGGGGTTATCGGTATGCG Deletion of *iscA* with *cat*

CAGCCTTTGAGTGAGCTGATAC

iscA-scr-1 CAAAGCCGCCATTGCG Screening of *iscA* deletion

iscA-scr-2 CTCAGGGCTGCGATCCG Screening of *iscA* deletion

ompF-KO-1 GTGGCAGGTGTCATAAAAAAAACCATGAGGGTAATA Deletion of *ompF* with *cat*

AATAGCTCATATGAATATCCTCCTTAG

ompF-KO-A GTGGCAGGTGTCATAAAAAAAACCATGAGGGTAATA Deletion of *ompF* with *kan*

AATATGTAGGCTGGAGCTGCTTC

ompF-KO-2 AAAGTCCTGTTTTTTCGGCATTTAACAAAGAGGTGTG Deletion of *ompF* with *cat/kan*

CTAGCCTTTGAGTGAGCTGATAC

ompF-scr-1 GACGGCAGTGGCAGGTG Screening of *ompF* deletion

ompF-scr-2 GACGTGAGATTGCTCTGGAAG Screening of *ompF* deletion
