# Supplementary material for: High-Throughput Proteomics Identifies Proteins With Importance to Postantibiotic Recovery in Depolarized Persister Cells
Source: Front Microbiol. 2019 Mar 6;10:378. doi: 10.3389/fmicb.2019.00378 (PMC6414554; doi:10.3389/fmicb.2019.00378)
Supplement: Supplementary file 4 [file Data_Sheet_1.PDF]

Supplementary Material

High-throughput proteomics identifies proteins with importance to postantibiotic recovery in depolarized persister cells

Daniel-Timon Spanka, Anne Konzer, Daniel Edelmann and Bork A. Berghoff*

*** Correspondence:** Dr. Bork A. Berghoff: Bork.A.Berghoff@mikro.bio.uni-giessen.de

Supplementary Figures

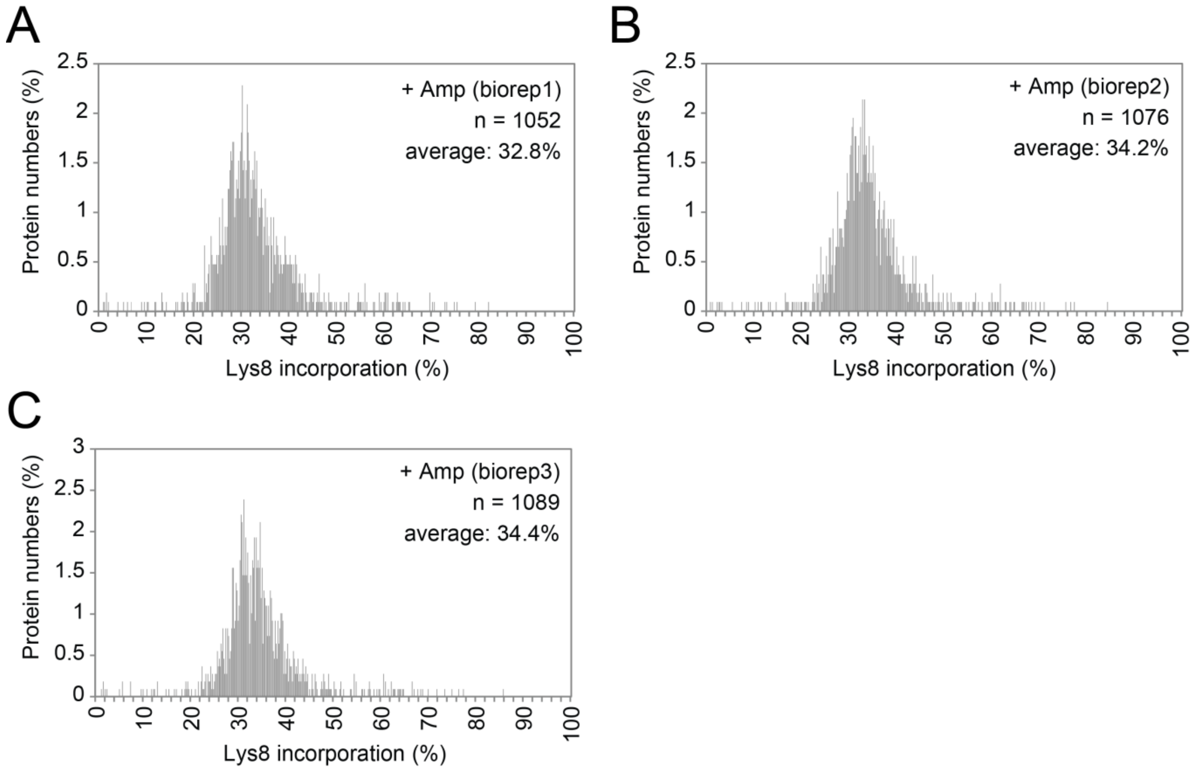

Supplementary Figure 1. Distribution plots of Lys8 incorporation in ampicillin-treated cultures. The plots show protein numbers (%) for any given Lys8 incorporation (%). The number of quantified proteins (n) and the average Lys8 incorporation are indicated for biorep1 (A), biorep2 (B), and biorep3 (C).

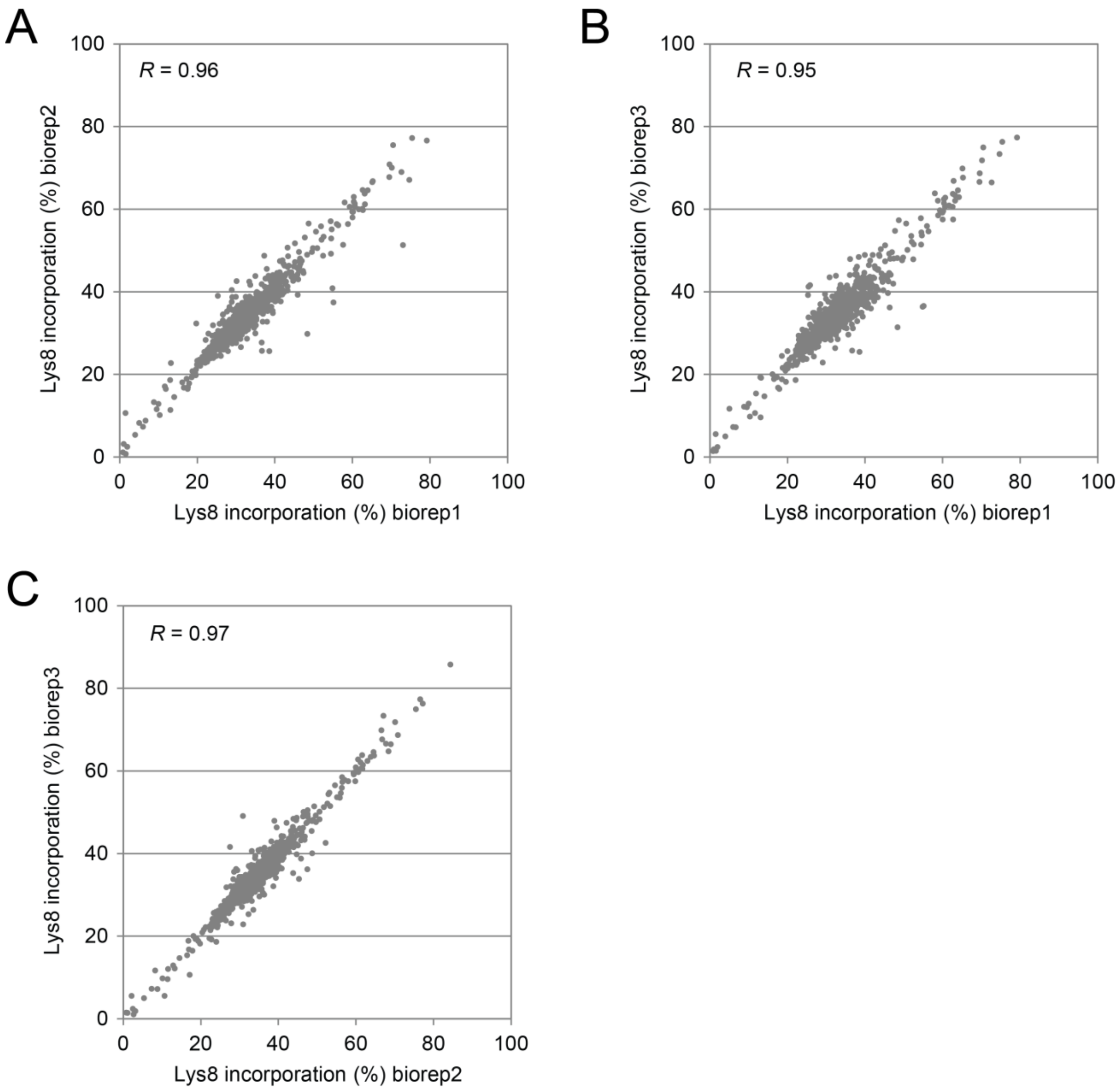

Supplementary Figure 2. Correlation plots of Lys8 incorporation in ampicillin-treated cultures. Lys8 incorporation (%) of individual proteins was compared between replicates. Comparisons are biorep1 versus biorep2 (A), biorep1 versus biorep3 (B), and biorep2 versus biorep3 (C). R = Pearson's Rho.

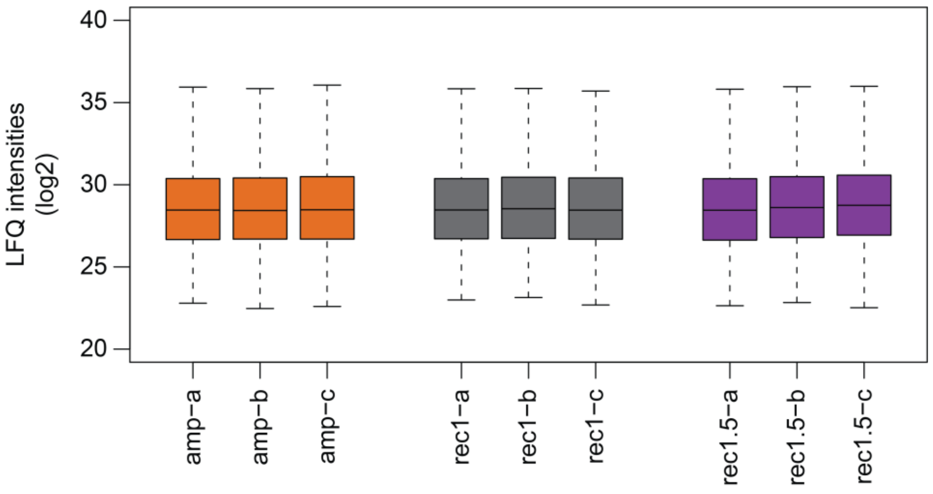

Supplementary Figure 3. Boxplots of log_2-transformed LFQ intensities. Label-free quantification (LFQ) intensities represent normalized protein intensities in the corresponding sample. Amp: two hours ampicillin treatment; rec1: one hour recovery; rec1.5: 1.5 hours recovery.

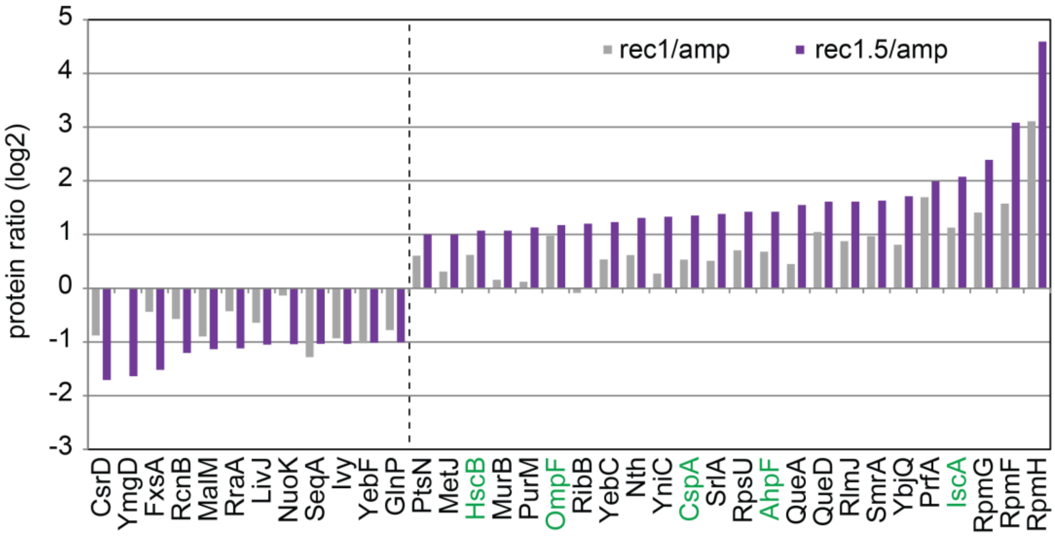

Supplementary Figure 4. Expression changes (log_2) between experimental conditions for 36 proteins that were identified as differentially expressed after 1.5 hours of recovery. Candidate proteins (see main text) are highlighted in green. Amp: two hours ampicillin treatment; rec1: one hour recovery; rec1.5: 1.5 hours recovery.

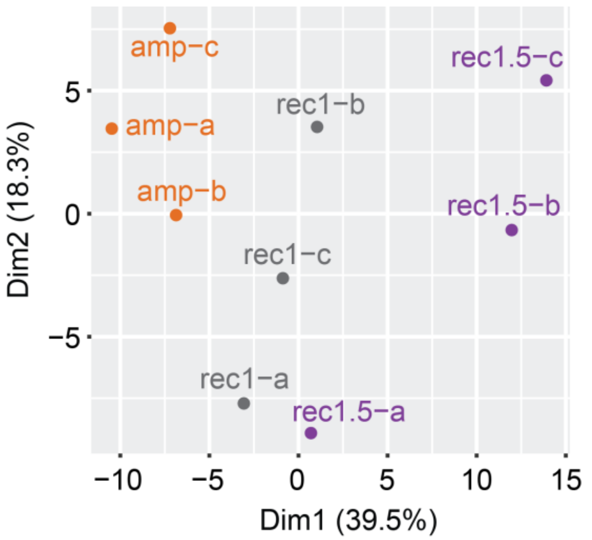

Supplementary Figure 5. Principal component analysis (PCA) of LFQ intensities. The plot is based on the two dimensions (Dim) explaining most of the variance between all samples. Amp: two hours ampicillin treatment; rec1: one hour recovery; rec1.5: 1.5 hours recovery.

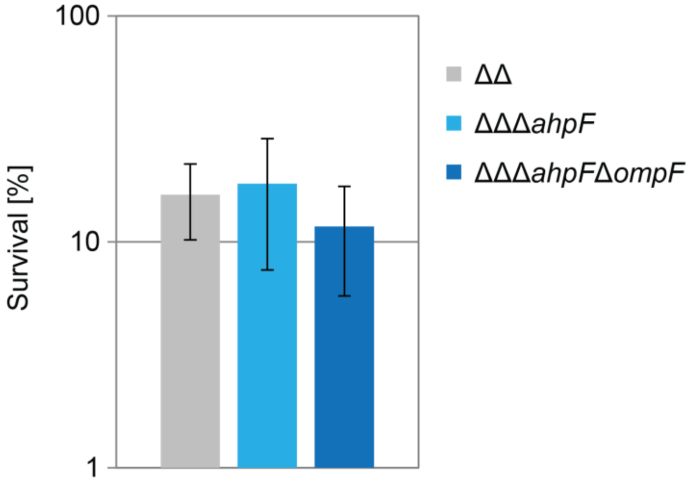

Supplementary Figure 6. Persister levels of strain Δ1-41 Δ*istR* (ΔΔ) and subsequent deletions of *ahpF* and *ompF*. Survival was calculated after three hours of ampicillin treatment (200 µg ml^{-1}) during exponential phase in liquid LB medium. Data represent the mean of at least three independent biological replicates. Error bars depict standard deviations.
